# Supplementary material for: Chemical reservoir computation in a self-organizing reaction network
Source: Nature. 2024 Jun 26;631(8021):549–55. doi: 10.1038/s41586-024-07567-x (PMC11254755; doi:10.1038/s41586-024-07567-x)
Supplement: Supplementary file 1 — This file contains Supplementary Figs. 1–21, extended mass spectrometry methods, further discussion of chemical reservoir computation and a further explanation of the various types of analysis used in this work. [file 41586_2024_7567_MOESM1_ESM.pdf]

nature portfolio

https://doi.org/10.1038/s41586-024-07567-x

# Supplementary information

# Chemical reservoir computation in a self-organizing reaction network

In the format provided by the authors and unedited

# Supplementary Information: Chemical reservoir computation in a self-organizing reaction network

Mathieu G. Baltussen[1], Thijs J. de Jong[1], Quentin Duez[1], William E. Robinson[1], and Wilhelm T.S. Huck[1*]

[1] *Institute for Molecules and Materials, Radboud University, Heyendaalseweg 135, 6525 AJ Nijmegen, The Netherlands*
[*] *email: w.huck@science.ru.nl*

# Contents


**1 Experimental methods** **S4**
1.1 Mass spectrometry analysis . . . S4
1.2 Data processing . . . S4

**2 Background** **S7**
2.1 Reservoir computation . . . S7
2.2 A theoretical example . . . S7
2.3 Training & testing . . . S8
2.4 *In chemico* reservoir computation . . . S9
2.5 Types of tasks . . . S9

**3 Nonlinear classification** **S11**
3.1 Data generation . . . S11
3.2 Reproducibility . . . S11
3.3 Classification tasks . . . S11
3.4 Training procedure . . . S13
3.5 Test scores by leave-5-out cross-validation . . . S13
3.6 Training set size . . . S13

**4 Dynamic systems predictions** **S14**
4.1 Fluctuating input & data generation . . . S14
4.2 Simulation of fluctuation-driven dynamical systems . . . S14
4.3 Training procedure . . . S14

**5 Forecasting** **S15**
5.1 Lorenz attractor input & data generation . . . S15
5.2 Training procedure . . . S15
5.3 Mutual information . . . S15

**References** **S17**


# List of Tables


1 List of assigned ion traces . . . S5

# Supplementary Figures


| | | |
|---|---|---|
| S1 | Ion alignment | S19 |
| S2 | Selected singly charged ions | S20 |
| S3 | Selected doubly charged ions | S21 |
| S4 | Selected doubly charged ions cont. | S22 |
| S5 | Steady state inputs and reservoir response | S23 |
| S6 | Steady state inputs and reservoir response (cont.) | S24 |
| S7 | Reproducibility of identical inputs | S25 |
| S8 | Scoring of Lasso classification results | S26 |
| S9 | Classification weights Ridge regression | S27 |
| S10 | Classification weights Lasso regression | S28 |
| S11 | Classification progress Lasso weights - 1 | S29 |
| S12 | Classification progress Lasso weights - 2 | S30 |
| S13 | Classification progress Lasso weights - 3 | S31 |
| S14 | Classification progress Lasso weights - 4 | S32 |
| S15 | Classification progress Lasso weights - 5 | S33 |
| S16 | Classification train-set size influence | S34 |
| S17 | Prediction results for substrates in the metabolic network | S35 |
| S18 | Prediction results for substrates in the metabolic network (cont.) | S36 |
| S19 | Normalized mean-squared errors for metabolic network predictions | S37 |
| S20 | Extended forecast time traces and comparison plots | S38 |
| S21 | Mutual information by compound | S38 |

# 1 Experimental methods

## 1.1 Mass spectrometry analysis

The diluted outflow of the CSTR was continuously injected in the electrospray ionization (ESI) source of a timsToF instrument (Bruker Daltonics, Bremen, Germany), whose ESI needle was replaced with a 10 cm long stainless steel tubing (0.2 mm outside diameter, 0.05 mm wall thickness, 0.1 mm inside diameter – Sigma Aldrich GF15490795). Ions were electrosprayed in positive mode with the following settings: Voltage +4.5 kV, nebulizer pressure 2.0 Bar, drying gas flow 8 L.min$^{-1}$, and source temperature set to 250°$C$.

Ions were then deflected towards the trapped ion mobility (TIMS) cell with a potential of 70 V. TIMS experiments were performed using $N_2$ as carrier gas by scanning ion mobility from 0.4 V.s/cm$^2$ to 0.84 V.s/cm$^2$. The ramp time was set to 500 ms, and the accumulation time to 20 ms in order to minimize ion activation in the ion mobility region. Delta potentials in the TIMS funnels were set as follows: $\Delta 1 = -20\ V$, $\Delta 2 = -150\ V$, $\Delta 3 = 50\ V$, $\Delta 4 = 0\ V$, $\Delta 5 = 0\ V$, $\Delta 6 = 0\ V$. RF potentials in the TIMS funnels were set as follows: Funnel 1 RF = 200 Vpp, Funnel 2 RF = 300 Vpp, Multipole RF = 100 Vpp.

Ion transfer voltages were Quadrupole Ion Energy = 1 eV, Collision Cell In = 150 V and Collision Energy = 3 eV. The ion transmission was optimized for the mass range of interest (m/z 80-300) by using a transfer time of 32 $\mu s$, a collision RF of 200 Vpp and a pre pulse storage time of 5 $\mu s$ prior to time-of-flight (ToF) analysis. The mass range scanned by the ToF analyser was m/z 50-650.

The MS and TIMS dimensions were calibrated linearly using three selected ions from the Agilent ESI LC/MS tuning mix [(118.0863, 0.542 V.s/cm$^2$),(322.0481, 0.732 V.s/cm$^2$), and (622.0290, 0.985 V.s/cm$^2$)].

## 1.2 Data processing

Raw timsToF data were loaded using the TimsPy and OpenTims libraries[1]. Ion counts were first normalized to the total ion current (TIC) to correct for eventual changes ion transmission over time. The complete dataset was then filtered to extract the intensities of 106 manually selected ions with unique $m/z$ and inverse mobilities ($1/K_0$) stored in a peak table. Ion intensities were extracted for the given $m/z$ with a mass width of 0.02 Da and given $1/K_0$ with a width of 0.006 V.s/cm$^2$. The peak table was established based on the most intense signals observed in several experiments and is shown in Table 1. The molecular composition of each ion was identified based on their precise mass.

To account for inverse mobility drift between days, a data rescaling was performed. The drift is quantified by comparing the apex of the mobilograms for two specific ions, $m/z$ 110.012 ($D_1$ - $[C_6H_{12}O_6Ca]^{2+}$) and 233.063 ($D_2$ - $[C_7H_{14}O_7Na]^+$), with a reference measurement ($R_1 = 0.518$ V.s/cm$^2$ and $R_2 = 0.683$ V.s/cm$^2$). This comparison is used to adjust inverse mobility values reported in the peak table ($R_x$) for drift in measurements, rather than realigning the drifted data itself (figure S1). The conversion of reference values to drifted values is performed with the following equation:

$$D_x = D_1 + (R_x - R_1)\left(\frac{D_2 - D_1}{R_2 - R_1}\right) \tag{1}$$

The ions constituting the peak table are reported in figures S2 to S4.

Table 1: **List of assigned ion traces** m/z values extracted with a mass width of 0.02 $Da$, and inverse mobility with a width of 0.006 V.s/cm$^2$

| **Composition** | $m/z$ | $1/K_0$ |
|---|---|---|
| $[C_4H_9O_4CaNa]^{2+}$ | 92.001 | 0.517 |
| $[C_5H_{10}O_5Ca]^{2+}$ | 95.007 | 0.521 |
| $[C_5H_{10}O_5Ca]^{2+}$ | 95.007 | 0.534 |
| $[C_2H_3O_2Ca]^{+}$ | 98.975 | 0.594 |
| $[C_2H_3O_2Ca]^{+}$ | 98.975 | 0.608 |
| $[C_6H_{10}O_5Ca]^{2+}$ | 101.007 | 0.518 |
| $[C_6H_{10}O_5Ca]^{2+}$ | 101.007 | 0.53 |
| $[C_6H_{12}O_6Ca]^{2+}$ | 110.012 | 0.519 |
| $[C_6H_{12}O_6Ca]^{2+}$ | 110.012 | 0.531 |
| $[C_6H_{12}O_6Ca]^{2+}$ | 110.012 | 0.542 |
| $[C_6H_{12}O_6Ca]^{2+}$ | 110.012 | 0.549 |
| $[C_6H_{12}O_6Ca]^{2+}$ | 110.012 | 0.558 |
| $[C_6H_{12}O_6Ca]^{2+}$ | 110.012 | 0.586 |
| $[C_7H_{14}O_6Ca]^{2+}$ | 117.02 | 0.521 |
| $[C_7H_{14}O_6Ca]^{2+}$ | 117.02 | 0.535 |
| $[C_7H_{14}O_6Ca]^{2+}$ | 117.02 | 0.553 |
| $[C_6H_{14}O_7Ca]^{2+}$ | 119.018 | 0.517 |
| $[C_6H_{14}O_7Ca]^{2+}$ | 119.018 | 0.532 |
| $[C_6H_{14}O_7Ca]^{2+}$ | 119.018 | 0.541 |
| $[C_6H_{14}O_7Ca]^{2+}$ | 119.018 | 0.559 |
| $[C_7H_{14}O_7Ca]^{2+}$ | 125.018 | 0.53 |
| $[C_7H_{14}O_7Ca]^{2+}$ | 125.018 | 0.542 |
| $[C_7H_{14}O_7Ca]^{2+}$ | 125.018 | 0.564 |
| $[C_7H_{16}O_7Ca]^{2+}$ | 126.026 | 0.522 |
| $[C_7H_{16}O_7Ca]^{2+}$ | 126.026 | 0.528 |
| $[C_7H_{16}O_7Ca]^{2+}$ | 126.026 | 0.54 |
| $[C_7H_{16}O_7Ca]^{2+}$ | 126.026 | 0.545 |
| $[C_7H_{16}O_7Ca]^{2+}$ | 126.026 | 0.565 |
| $[C_6H_{16}O_8Ca]^{2+}$ | 128.023 | 0.517 |
| $[C_6H_{16}O_8Ca]^{2+}$ | 128.023 | 0.534 |
| $[C_6H_{16}O_8Ca]^{2+}$ | 128.023 | 0.541 |
| $[C_6H_{16}O_8Ca]^{2+}$ | 128.023 | 0.56 |
| $[C_3H_5O_3Ca]^{+}$ | 128.985 | 0.624 |
| $[C_7H_{16}O_8Ca]^{2+}$ | 134.023 | 0.53 |
| $[C_7H_{16}O_8Ca]^{2+}$ | 134.023 | 0.537 |
| $[C_7H_{16}O_8Ca]^{2+}$ | 134.023 | 0.564 |
| $[C_7H_{16}O_8Ca]^{2+}$ | 134.023 | 0.571 |
| $[C_9H_{18}O_7Ca]^{2+}$ | 139.033 | 0.527 |
| $[C_9H_{18}O_7Ca]^{2+}$ | 139.033 | 0.534 |
| $[C_9H_{18}O_7Ca]^{2+}$ | 139.033 | 0.543 |
| $[C_9H_{18}O_7Ca]^{2+}$ | 139.033 | 0.567 |
| $[C_8H_{16}O_8Ca]^{2+}$ | 140.023 | 0.525 |
| $[C_8H_{16}O_8Ca]^{2+}$ | 140.023 | 0.54 |
| $[C_8H_{16}O_8Ca]^{2+}$ | 140.023 | 0.551 |
| $[C_8H_{16}O_8Ca]^{2+}$ | 140.023 | 0.575 |
| $[C_8H_{18}O_8Ca]^{2+}$ | 141.031 | 0.529 |
| $[C_8H_{18}O_8Ca]^{2+}$ | 141.031 | 0.538 |
| $[C_8H_{18}O_8Ca]^{2+}$ | 141.031 | 0.572 |
| $[C_7H_{18}O_9Ca]^{2+}$ | 143.028 | 0.535 |
| $[C_7H_{18}O_9Ca]^{2+}$ | 143.028 | 0.544 |
| $[C_7H_{18}O_9Ca]^{2+}$ | 143.028 | 0.572 |
| $[C_9H_{18}O_8Ca]^{2+}$ | 147.031 | 0.531 |
| $[C_9H_{18}O_8Ca]^{2+}$ | 147.031 | 0.539 |
| $[C_9H_{18}O_8Ca]^{2+}$ | 147.031 | 0.545 |
| $[C_9H_{18}O_8Ca]^{2+}$ | 147.031 | 0.575 |
| $[C_8H_{18}O_9]^{2+}$ | 149.028 | 0.537 |
| $[C_8H_{18}O_9]^{2+}$ | 149.028 | 0.545 |
| $[C_8H_{18}O_9]^{2+}$ | 149.028 | 0.551 |
| $[C_8H_{18}O_9]^{2+}$ | 149.028 | 0.579 |
| $[C_9H_{18}O_9Ca]^{2+}$ | 155.028 | 0.542 |
| $[C_9H_{18}O_9Ca]^{2+}$ | 155.028 | 0.548 |
| $[C_9H_{18}O_9Ca]^{2+}$ | 155.028 | 0.558 |
| $[C_9H_{18}O_9Ca]^{2+}$ | 155.028 | 0.565 |
| $[C_4H_7O_4Ca]^{+}$ | 158.996 | 0.625 |
| $[C_4H_7O_4Ca]^{+}$ | 158.996 | 0.642 |
| $[C_9H_{20}O_{10}Ca]^{2+}$ | 164.033 | 0.549 |
| $[C_9H_{20}O_{10}Ca]^{2+}$ | 164.033 | 0.559 |
| $[C_9H_{20}O_{10}Ca]^{2+}$ | 164.033 | 0.577 |
| $[C_9H_{20}O_{10}Ca]^{2+}$ | 164.033 | 0.587 |
| $[C_{10}H_{20}O_{10}Ca]^{2+}$ | 170.033 | 0.558 |
| $[C_{10}H_{20}O_{10}Ca]^{2+}$ | 170.033 | 0.565 |
| $[C_{10}H_{20}O_{10}Ca]^{2+}$ | 170.033 | 0.584 |
| $[C_{10}H_{20}O_{10}Ca]^{2+}$ | 170.033 | 0.594 |
| $[C_5H_{10}O_5Na]^{+}$ | 173.041 | 0.623 |
| $[C_5H_{10}O_5Na]^{+}$ | 173.041 | 0.636 |
| $[C_{10}H_{22}O_{11}Ca]^{2+}$ | 179.039 | 0.566 |
| $[C_{10}H_{22}O_{11}Ca]^{2+}$ | 179.039 | 0.573 |
| $[C_{10}H_{22}O_{11}Ca]^{2+}$ | 179.039 | 0.596 |
| $[C_{11}H_{24}O_{11}Ca]^{2+}$ | 185.039 | 0.572 |
| $[C_{11}H_{24}O_{11}Ca]^{2+}$ | 185.039 | 0.602 |
| $[C_6H_{10}O_5Na]^{+}$ | 185.039 | 0.619 |
| $[C_6H_{10}O_5Na]^{+}$ | 185.039 | 0.639 |
| $[C_6H_{10}O_5Na]^{+}$ | 185.039 | 0.651 |
| $[C_6H_{10}O_5Na]^{+}$ | 185.039 | 0.673 |
| $[C_{11}H_{24}O_{12}Ca]^{2+}$ | 194.044 | 0.57 |
| $[C_{11}H_{24}O_{12}Ca]^{2+}$ | 194.044 | 0.577 |
| $[C_{11}H_{24}O_{12}Ca]^{2+}$ | 194.044 | 0.604 |
| $[C_7H_{12}O_5Na]^{+}$ | 199.057 | 0.635 |
| $[C_7H_{12}O_5Na]^{+}$ | 199.057 | 0.647 |
| $[C_7H_{12}O_5Na]^{+}$ | 199.057 | 0.657 |
| $[C_7H_{12}O_5Na]^{+}$ | 199.057 | 0.667 |

| **Composition** | ***m/z*** | $1/K_0$ |
|---|---|---|
| $[C_7H_{12}O_5Na]^+$ | 199.057 | 0.68 |
| $[C_7H_{12}O_5Na]^+$ | 199.057 | 0.694 |
| $[C_{12}H_{24}O_{12}Ca]^{2+}$ | 200.044 | 0.575 |
| $[C_{12}H_{24}O_{12}Ca]^{2+}$ | 200.044 | 0.587 |
| $[C_{12}H_{24}O_{12}Ca]^{2+}$ | 200.044 | 0.61 |
| $[C_6H_{12}O_6Na]^+$ | 203.052 | 0.655 |
| $[C_6H_{12}O_6Na]^+$ | 203.052 | 0.663 |
| $[C_6H_{12}O_6Na]^+$ | 203.052 | 0.67 |
| $[C_6H_{12}O_6Na]^+$ | 203.052 | 0.676 |
| $[C_{12}H_{26}O_{13}Ca]^{2+}$ | 209.05 | 0.577 |
| $[C_{12}H_{26}O_{13}Ca]^{2+}$ | 209.05 | 0.583 |
| $[C_{12}H_{26}O_{13}Ca]^{2+}$ | 209.05 | 0.613 |
| $[C_7H_{14}O_7Na]^+$ | 233.063 | 0.683 |
| $[C_7H_{14}O_7Na]^+$ | 233.063 | 0.693 |
| $[C_7H_{14}O_7Na]^+$ | 233.063 | 0.7 |

# 2 Background

## 2.1 Reservoir computation

Reservoir computation is a neuromorphic computation paradigm, in which a (physical) system is used as a type of black-box artificial neural network[2]. This network responds in a nonlinear fashion to any inputs it receives, after which a final output or computational answer is obtained by taking an appropriate linear combination of readout nodes in the network. Training only takes place for the readout weights, often using a simple linear regression. By directly employing the inherent complexity and capacity of the reservoir for information processing, expensive training of any internal weights can be circumvented, while still allowing for remarkably accurate calculations. It has been shown that sufficiently complex reservoirs hold a similar computational power to regular neural networks, where all interaction parameters need to be tuned/trained, thus lowering the computational and energy costs for training such networks to specific problems[3]. The principles of reservoir computation were first shown to be effective neural networks through the (virtual) creation of liquid-state machines [4] and echo-state networks [5].

For a physical system to be an effective reservoir, it must have the following properties[2]:

1. A high-dimensional internal state space
2. Nonlinear interactions between nodes
3. Fading memory
4. Sufficient separation and generalizability of input signals

A wide variety of systems have been designed and created, based on the same overarching principles of reservoir computation. Examples of this include cellular automata [6], electronic [7] photonic [8] and spintronic circuits [9], soft bodies [10], and *in vitro* cell cultures [11].

## 2.2 A theoretical example

Traditionally, a reservoir computer is described as a dynamical system with internal variables $\mathbf{x}$ that change over time as

$$\frac{d\mathbf{x}}{dt} = g(\mathbf{x}) \tag{2}$$

This dynamical system is connected to some inputs $\mathbf{u}$ which additionally may be time-dependent (e.g. $\mathbf{u}(t)$). This results in a dynamical system of the form

$$\frac{d\mathbf{x}}{dt} = g(\mathbf{x}) + \mathbf{u}(t) \tag{3}$$

Let's now assume we want to use the reservoir to approximate (or *compute*) a random function $f$. This function can essentially denote any kind of desired computation, such as analytical expressions, integral solutions of differential equations, chaotic mappings, and so on. In general, the function takes some input denoted by $u$ and produces an output denoted by $y$, where both can be time-dependent, e.g.

$$\mathbf{y}(t) = f(\mathbf{u}(t)) \tag{4}$$

Using the reservoir, we can approximate any function $f$ as follows: any input $\mathbf{u}(t)$ produces a complex reservoir response $\mathbf{x}(t)$. This reservoir response can be transformed into a computational output by multiplication with a weight vector (or matrix) $W$ to obtain $\hat{\mathbf{y}}(t) = W \cdot \mathbf{x}(t)$. By choosing appropriate values for the weights, we can ensure that the computational output $\hat{\mathbf{y}}(t)$ approximates the output $\mathbf{y}(t)$ of the function $f$, such that

$$f(\mathbf{u}(t)) = \mathbf{y}(t) \approx \hat{\mathbf{y}}(t) = W \cdot \mathbf{x}(t) \tag{5}$$

Note here that the final step of converting the reservoir response into some computational output is completely linear. Applying weights essentially allows us to choose and combine parts of the reservoir which have a (combined) response similar to function that we desire to approximate. However, the appropriate types of responses need to exist within the reservoir's dynamics for it to be able to approximate a desired function. Therefore, we can expect that not every reservoir can perform every type of computation.

## 2.3 Training & testing

In physical reservoir computation the only part of the computation that is done *in silico* is calculation of the weights $W$. While this calculation is in principle a simple task, different strategies exist to determine weights that are more robust to experimental noise, outliers, and so on.

Generally, weights are obtained by minimizing a distance $d(\mathbf{y}_{train}(t), W \cdot \mathbf{x}_{train}(t))$ between the computational response $\mathbf{x}(t)$ of the reservoir and the true function output $\mathbf{y}(t)$ for some training set where the true function outputs are already known, e.g.

$$\arg\min_{W} d(\mathbf{y}_{train}(t), W \cdot \mathbf{x}_{train}(t)) \tag{6}$$

However, depending on the type of function and the type of data an alternative distance measure may be used, such as the Euclidean distance

$$d_2(\mathbf{y}, W\mathbf{x}) = \sqrt{\mathbf{y}^2 - (W\mathbf{x})^2} \tag{7}$$

a higher-order distance

$$d_n(\mathbf{y}, W\mathbf{x}) = (\mathbf{y}^n - (W\mathbf{x})^n)^{1/n} \tag{8}$$

or even a Gaussian kernel

$$d_\sigma(\mathbf{y}, W\mathbf{x}) = e^{-(\mathbf{y}^2-(W\mathbf{x})^2)/\sigma^2} \tag{9}$$

Furthermore, if $\mathbf{x}$ is very high-dimensional - e.g. the reservoir has many observable components - a penalized minimization strategy can be used to promote a sparser set of weights and reduce variability in the computational output. This is especially useful when multiple observable components of the reservoir are collinear, essentially showcasing similar nonlinear behaviour. Examples of strategies like this are lasso regression—also known as $L1$ regularization—where the minimization problem is modified to

$$\arg\min_{W} d_2(\mathbf{y}, W\mathbf{x}) + \lambda|W| \tag{10}$$

and ridge regression—also known as $L2$ regularization—which results in

$$\arg\min_{W} d_2(\mathbf{y}, W\mathbf{x}) + \lambda W^2 \tag{11}$$

Lasso regressions will generally result in a sparse weight matrix, meaning that only a select number of features are being selected, while the weights of many features will be set to zero. Ridge regression will result in many features being used while keeping all the weights as small as possible, which helps mitigate the problem of collinearity between features.

## 2.4 *In chemico* reservoir computation

Chemical reaction networks have the potential to incorporate the various properties necessary for reservoir computation. In non-selective chemical settings, highly complex and strongly branched chemical reaction networks may emerge. Especially in networks with recursive chemical reactions, a combinatorial explosion of unique molecules may be created, leading to an increasingly high-dimensional internal state space. Generally, many of the chemical reactions in such systems are between different compounds, potentially catalyzed by the presence of a catalyst. This leads to interactions that vary from linear, for unimolecular reactions, to strongly nonlinear, for reactions occurring via complex catalyzed reaction schemes. These properties may allow some chemical reaction networks the necessary complexity for reservoir computation.

However, for the reservoir to be able to process a series of different inputs, a fading memory of those inputs is required. This property can be physically achieved by situating the chemical reaction network in an out-of-equilibrium setting. The exchange of compounds with an external environment firstly allows the system to receive inputs in the form of different compounds, but also to have the effect of those inputs to disappear over time by the outflow of compounds. Various techniques exist to bring, and keep, chemical reaction networks out-of-equilibrium. In this work we achieve this by using continuously-stirred flow reactors, which can continuously receive new inputs, while flushing out the reactor content over time. The inflow and outflow of compounds can be effectively modelled as an additional flow-term in the ordinary differential equations describing the dynamics of the chemical reaction network[12]. The effect of the flow reactor on the dynamics of the chemical reaction network are essentially equivalent to the fading-memory property as often encoded in reservoir computers (and continuous-time recursive neural networks in general). This can be seen directly from the form of the differential equations describing a general chemical reaction network in flow:

$$\frac{d[C]]}{dt} = g([C]) + k_f[u] - k_f[C] \tag{12}$$

with $k_f$ the flowrate, $[C]$ the concentration of chemical species, $[u]$ the input flown into the reactor, and $g([C])$ a general description of the reaction kinetics (which can often be approximated using a mass-action kinetics framework). Here, the term $k_f[C]$ establishes behaviour similar to that of fading memory nodes in, for example, Hopfield networks[13] and various reservoir computers[14]. Specifically, chemical species $[C]$ that are not directly connected to an input (e.g. $u = 0$) and are also kinetically separated from input species (e.g. the interactions are slow or take multiple steps) can be interpreted and employed as more robust, but less responsive, memory nodes.

## 2.5 Types of tasks

In this work, we make a distinction between 3 specific types of computational tasks that can be solved using the reservoir computation approach. These are i) analytical expressions, ii) integral solutions of non-homogeneous ordinary differential equations, and iii) chaotic maps. All three types are briefly (and non-formally) discussed below to explain their underlying logic.

### Analytic expressions

Analytical expressions are commonly understood to be all mathematical expressions that can be written down and solved in *closed form*. This includes constants, arithmetic functions $(+, -, x, -)$, powers and roots $(x^n, x^{1/n})$, as well as exponential functions $(e^{ax})$, logarithms $(\ln x)$ and trigonometric functions $(\sin(x), \cos(x), \dots)$, as well as more specialized functions and series expansion, such as the Bessel functions and convergent summations. We also include classifications in this definition, as they can be represented by any of the above operations together with the modulus $(|x|)$ or modulo $(x mod n)$ operators.

For computation of such functions using the reservoir computer approach, this implies that the output should only be dependent on a singular input, and should not be influenced by any historical inputs that the reservoir may have received. Generally, this means that these types of functions are only calculated using static inputs and steady-state data.

### Integral solutions of non-homogeneous ordinary differential equations

Next, the reservoir can also approximate the behaviour of differential equations when exposed to a time-dependent external driving force, which we call the input $\mathbf{u}(t)$. These are non-homogeneous ordinary differential equations of the form

$$\frac{d\mathbf{y}}{dt} = g(\mathbf{y}, \mathbf{u}(t)) \tag{13}$$

Solutions to these type of equations are normally obtained by integration as

$$y(t) = f(\mathbf{u}(t)) = \int_{t_0}^{t} g(\mathbf{y}, \mathbf{u}(\tau))d\tau \tag{14}$$

The solutions to this equation depend not just on the form of the equation $g$, but also on the initial state $y_0 = y(t_0)$ and input (or driving force) over time $\mathbf{u}(t)$. Thus, solving this equation for a specific $u(t)$ requires knowledge about past values of $u(t)$ as well as current values, in contrast to the analytical expressions discussed above.

For computation of the function $y(t) = f(\mathbf{u}(t))$, the reservoir is required to retain some information from its past input in order to yield a result similar to the integration.

### Chaotic maps

The third category of functions to compute in this work is the forecasting of chaotic dynamics. We now assume that the input to the reservoir $\mathbf{u}(t)$ represents a solution to a chaotic system (such as the Lorenz attractor equations, as shown in 5). This means that $\mathbf{u}(t)$ evolves according to a differential (or difference in discrete cases) equation that is sensitive to initial conditions (similar current trajectories will diverge over time), non-periodic, and shows topological mixing.

In contrast to most dynamical systems, forecasting the behaviour of a chaotic system is intrinsically difficult, as it requires estimating a function of the form

$$\mathbf{u}(t + \delta t) = f(\mathbf{u}(t)) \tag{15}$$

that is extremely sensitive to the specific state of $\mathbf{u}(t)$ as well as previous states, and is therefore often highly non-linear and non-trivial.

# 3 Nonlinear classification

Nonlinear classification of two-dimensional data is a standard class of supervised learning problems. It specifically refers to problems that cannot be effectively solved using a linear classifier, such as linear regression or a linear support-vector classifier (LSVC). We focus on a selection of nonlinear classification tasks, showcasing a broad range of applicability. All tasks start with the same two-dimensional input of specific NaOH and formaldehyde concentrations. These inputs can be appropriately rescaled and recentred for each specific task.

Specifically, we want to perform a classification of inputs $\mathbf{u_i}$ sampled from a two-dimensional input space $\mathcal{U} = [0, 1] \times [0, 1]$. The set of inputs $\{\mathbf{u_i}\}$ is sampled using a Latin hypercube algorithm[15] to ensure a uniform sampling space. For every classification task, a nonlinear function $f$ exists that for every input $\mathbf{u_i}$ assigns an outcome (or label) $y_i = f(\mathbf{u_i})$.

## 3.1 Data generation

To perform the nonlinear classification with the formose reservoir computer, the inputs $\mathbf{u_i}$ are first transformed to a range of appropriate chemical inflow concentrations, with the first component of the input linearly mapped to the NaOH $[10, 50]$ $mM$ concentration range, and the second component linearly mapped to the formaldehyde $[10, 150]$ $mM$ concentration range. The DHA and $CaCl_2$ inputs were held constant at 50 $mM$ and 15 $mM$ respectively. Due to experimental (time) limitations, the full set of inputs was split into five different sets, for which the FRC response was measured on different days. These five different sets were then separated further into two sets covering either the bottom or top half of the formaldehyde input concentration range. These partitions are shown in figures S5 and S6, with the inputs per experiment shown on the left, and the resulting ion signals on the right. Repeat-inputs were included at the start, end, and during every experiment run to check reproducibility of the measured output (see section 3.2). The reservoir output was measured by ion mobility-mass spectrometry, as the relative abundance of specific ions following the procedure detailed in section 1.2.

Every input was applied to the system for a duration of 30 minutes, to ensure that the FRC reached a steady-state. The outputs were read during the last 10 minutes (600 seconds) for each 30 minutes period, and were then used as steady-state data, averaging over the observed signal intensities to reduce noise. Finally, for every input we collect the averaged steady-state output results in a 106-dimensional vector, to obtain the chemical output as a function of input $\mathbf{x}_i = g(\mathbf{u_i})$, where $g$ denotes the response of the reservoir.

## 3.2 Reproducibility

To determine how reproducible the data is across different experimental days identical inputs were measured consistently across each nonlinear classification experiment. The relative intensities and corresponding error bars are shown in figure S7. This demonstrates that the relative error is enough to clearly differentiate between different compounds, across days and conditions.

## 3.3 Classification tasks

We determined the nonlinear classification capabilites of the FRC by attempting 10 different classification tasks, which are detailed below:

### AND gate

$$AND(x, y) = \begin{cases} 1 \text{ if } x > 0.5 \wedge y > 0.5 \\ 0 \text{ otherwise} \end{cases} \tag{16}$$

### OR gate

$$OR(x,y) = \begin{cases} 0 \text{ if } x < 0.5 \wedge y < 0.5 \\ 1 \text{ otherwise} \end{cases} \tag{17}$$

### Linear separator

$$Linear(x,y) = \begin{cases} 1 \text{ if } y > x \\ 0 \text{ otherwise} \end{cases} \tag{18}$$

### Triangular separator

$$Triangle(x,y) = \begin{cases} 1 \text{ if } (y > x/3 + 1/3) \wedge (x < y/3 + 1/3) \\ 0 \text{ if } (y < x/3 + 1/3) \wedge (x < y/3 + 1/3) \\ -1 \text{ otherwise} \end{cases} \tag{19}$$

### XOR gate

$$XOR(x,y) = \begin{cases} 1 \text{ if } (x > 0.5 \wedge y < 0.5) \vee (x < 0.5 \wedge y > 0.5) \\ 0 \text{ otherwise} \end{cases} \tag{20}$$

### Checkers pattern

$$XOR(x,y) = \begin{cases} 1 \text{ if } (\lfloor 3x \rfloor \bmod 2 \wedge \lfloor 3y \rfloor \bmod 2) \vee (\lfloor 3x \rfloor \bmod 2 \wedge \lfloor 3y \rfloor \bmod 2) \\ 0 \text{ otherwise} \end{cases} \tag{21}$$

### Circle

$$Circle(x,y) = \begin{cases} 1 \text{ if } ((x-0.5)^2 + (y-0.5)^2 < 0.125) \\ 0 \text{ otherwise} \end{cases} \tag{22}$$

### Sine wave

$$Sine(x,y) = \begin{cases} 1 \text{ if } y > 0.5 + 0.5 * \sin \frac{2\pi x}{0.8} - \frac{\pi}{4} \\ 0 \text{ otherwise} \end{cases} \tag{23}$$

### Concentric circles

$$Concentric(x,y) = \begin{cases} 1 \text{ if } ((x-0.5)^2 + (y-0.5)^2 < 0.16) \wedge ((x-0.5)^2 + (y-0.5)^2 > 0.04) \\ 0 \text{ otherwise} \end{cases} \tag{24}$$

### Dots

$$Dots(x,y) = \begin{cases} 1 \text{ if } ((x-0.3)^2 + (y-0.3)^2 < 0.05) \\ 1 \text{ if } ((x-0.7)^2 + (y-0.7)^2 < 0.05) \\ 0 \text{ otherwise} \end{cases} \tag{25}$$

### 3.4 Training procedure

Following the standard reservoir computer approach[3], we now train a linear regression model $y_i = h(x_i)$ that maps the chemical outputs to the classification results. Specifically, for the discrete nonlinear classification tasks we employ a linear support vector machine (LSVM) to better accommodate the transition between the continuous nature of the chemical output, and the discrete nature of the desired outcomes. $L_2$-regularization is used to obtain the weights during training in order to counteract the high collinearity between some ions in the chemical output.

### 3.5 Test scores by leave-5-out cross-validation

To test the prediction capabilities of the FRC for nonlinear classification tasks, we perform a stratified leave-5-out cross validation. In this procedure, during training of the LSVM we randomly leave out 5 input-output pairs such that we preserve the percentage of samples for each class. After training, we apply the LSVM to the 5 left-out chemical outputs to obtain 5 predictions $\hat{y}_j$, for which we calculate Matthew's Correlation Coefficient (MCC, also called the $\phi$-coefficient):

$$\phi = \frac{TP \times TN - FP \times FN}{\sqrt{(TP + FP)(TP + FN)(TN + FP)(TN + FN)}} \tag{26}$$

where $TP$ denotes the number of True Positives, $TN$ denotes True Negatives, $FP$ denotes False positives, and $FN$ denotes False Negatives. The MCC ranges between -1 and $+1$, with $+1$ corresponding to perfect predictions and -1 corresponding to predictions that are all wrong. For a classification task with two categories of equal size, a score of 0 corresponds to random guessing. The MCC is generally considered the most reliable and interpretable measure for classification task accuracies[16]. We have further calculated the $\phi$-accuracy of every task as

$$\phi \text{ acc.} = \frac{\phi + 1}{2} \tag{27}$$

This procedure is repeated for 20 repeats of 26 leave-5-out train-test splits, 520 repeats in total, after which all MCC-scores are averaged to obtain a leave-5-out cross validation (L5O-CV) score.

### 3.6 Training set size

To further investigate at what size the dataset is capable of achieving reliable and robust predictions, an additional cross-validation was performed on random train-test splits of the full datasets for increasing train set sizes. For every dataset size, 520 random train-test splits (stratified) were performed and the resulting training and testing scores were averaged.

The resulting learning curves are shown in figure S16.

# 4 Dynamic systems predictions

## 4.1 Fluctuating input & data generation

Noisy DHA flow profiles were generated usingnumpy. Values were generated using a normal distribution around the base flow value of 36.25 $\mu L/min$ with a standard deviation of 36.26/3.5 $\mu L/min$, tuned such that there are no negative values generated while keeping the flow rate as large as possible. These flowrates correspond to a mean concentration of 50 $mM$ with a standard deviation of $\sim$ 14.268 $mM$. Each flow rate was held constant for 60 seconds, before switching. The formaldehyde, NaOH and $CaCl_2$ inputs were held constant at 50 $mM$, 30 $mM$ and 15 $mM$ respectively.

## 4.2 Simulation of fluctuation-driven dynamical systems

For the *in silico* simulation, a network of the carbon-metabolism of E. Coli[17] was adapted. This was done by modifying the SBML file of that publication on the BioModels archive (`https://www.ebi.ac.uk/biomodels/MODEL2010160002`) using the Tellurium Python package, such that all metabolites in the network had additional inflow and outflow terms of the form $\varnothing \xrightarrow{k_f X_{in}} X$ and $X \xrightarrow{k_f X} \varnothing$. The flow constant was set to $k_f = 0.5\text{min}^{-1}$, and the inflow concentrations $X_{in}$ were set to the initial concentrations of the model. This modified SBML file was subsequently compiled into a C++ module by the Amici computational package [18] and loaded as a Python module.

To generate the training and test sets, the model was first run for 1000 minutes until steady-state was reached. Then, for every step in the fluctuating input pattern (every 60 seconds), the DHA input flow concentration was set to the corresponding value of the physical flow profile. The model was simulated with this input flow for the duration of the physical flow profile (1 minute) before the new DHA input flow was set. For every step, the simulation was initialized at the final state of the previous step. By appending the results of all simulation steps, a complete record of the behaviour of the network under fluctuating conditions was obtained.

## 4.3 Training procedure

Next, we trained the recorded formose reservoir response (binned in 10 second intervals to reduce noise) on the individual substrate time series of the model. This was done using a ridge regression algorithm with the regularization strength set to $\alpha = 5 \cdot 10^{-5}$. The predictions were scored using the normalized mean-squared error (NMSE):

$$\text{NMSE} = \frac{\sum_i (\hat{y}_i - y_i)^2}{\sum_i (y_i - \bar{y}_i)^2} \tag{28}$$

with $y$ the true values, $\bar{y}$ true value averaged over time, $\hat{y}$ the predicted values by the formose reservoir, and $i$ the index running over all substrates.

Extended results of this process are shown in figures S17 and S18 and NMSE calculations in figure S19

# 5 Forecasting

## 5.1 Lorenz attractor input & data generation

Flow profiles were generated using equation 29, using standard conditions of $\rho = 28.0$, $\beta = 8/3$, $\sigma = 10.0$, with an initial state of 10 for each axis. The x, y and z axis were assigned to NaOH, DHA and formaldehyde respectively.

$$\begin{aligned} \frac{dx}{dt} &= \sigma(y - x) \\ \frac{dy}{dt} &= x(\rho - z) - y \\ \frac{dz}{dt} &= xy - \beta z \end{aligned} \tag{29}$$

The system was solved using scipy.integrate.odeint, varying t from 0 to 80 with a total of 6480 steps. Each output step was matched to one second in the flow profile. A different scaling factor was used for each compound, 1.4, 1 and 1.3 for NaOH, DHA and formaldehyde respectively. The base flow rate is 30.2083 $\mu L/min$ for each syringe, with base concentrations of NaOH, DHA and formaldehyde at 30 $mM$, 50 $mM$ and 50 $mM$ respectively. The $CaCl_2$ concentration was held constant at 15 $mM$.

## 5.2 Training procedure

Next, we trained the recorded formose reservoir response (binned in 10 second intervals to reduce noise) on the same Lorenz attractor input 2 minutes into the future, using as target function $f(\mathbf{u}) = \mathbf{u}(t + 2min.)$. This was done using a ridge regression algorithm with the regularization strength set to $\alpha = 5 \cdot 10^{-5}$ on 30 minutes of the recorded formose output, after which inputs were successfully forecast 2 minutes ahead for the next 3 hours of data (fig. S20).

## 5.3 Mutual information

Generally, the FRC shows a nonlinear response to the chemical inputs it receives. We want to quantify which parts of the FRC, e.g. which compounds, contribute most to the processing of these inputs, and if distinct parts process inputs differently. Because the relationship between input and output(s) may be non-linear, standard correlation methods may not capture the full interaction picture. Instead, we choose to quantify the input-output relationships in terms of mutual information. Mutual information quantifies directly the 'amount of information' that can be obtained about one variable by observing the other variable, which in the case of the FRC correspond respectively to input and (a) output. Because MI is an information-theoretic quantity, it is not limited to linear dependencies, making it highly suitable for the nonlinear effects in the context of *in chemico* reservoir computation.

Mutual information is defined for a pair of random variables $X$ and $Y$ as

$$I(X;Y) = D_{KL}(P_{(X,Y)} || P_X \otimes P_Y) \tag{30}$$

$$= \int_Y \int_X P_{(X,Y)}(x,y) \log\left(\frac{P_{(X,Y)}(x,y)}{P_X(x)P_Y(y)}\right) \tag{31}$$

with $P_X$ and $P_Y$ the marginal distributions, and $P_{(X,Y)}$ the joint distribution of the random variables. The MI calculates the Kullback-Leibler divergence (a type of statistical distance), between the observed joint probability distribution of both variables and the hypothetical joint probability if both variables were independent (given by the product of marginal distribution).

Depending on the base of the logarithm, this quantity is either expressed in *Shannon* (base 2, also known as the *bit*), the *nat* (base $e$), or the *Hartley* (base 10). In this paper, mutual information uses the natural logarithm and is therefore given in *nat*s.

In practice, the MI can only be calculated by first estimating the probability distributions $P_X$ and $P_Y$ associated to the random variables, as only a finite number of observations of $X$ and $Y$ may be obtained. These estimates are often obtained by appropriate binning of the observations, but estimations based on entropy-calculations of k-nearest-neighbours has also been shown to work[19]. The second method is used in this paper, using a standard implementation provided by the Scikit-learn computational package[20] in Python.

In figure S21 the mutual information between every ion signal and respectively DHA, NaOH and formaldehyde inputs (as described in the previous section) are shown.

# References

1. Łącki, M. K., Startek, M. P., Brehmer, S., Distler, U. & Tenzer, S. OpenTIMS, TimsPy, and TimsR: Open and Easy Access to timsTOF Raw Data. *Journal of Proteome Research* **20,** 2122–2129 (2021).
2. Tanaka, G. *et al.* Recent advances in physical reservoir computing: A review. *Neural Networks* **115,** 100–123 (2019).
3. Lukoševičius, M. & Jaeger, H. Reservoir computing approaches to recurrent neural network training. *Computer Science Review* **3,** 127–149 (2009).
4. Maass, W., Natschläger, T. & Markram, H. Real-Time Computing Without Stable States: A New Framework for Neural Computation Based on Perturbations. *Neural Computation* **14,** 2531–2560 (2002).
5. Jaeger, H. Harnessing Nonlinearity: Predicting Chaotic Systems and Saving Energy in Wireless Communication. *Science* **304,** 78–80 (2004).
6. Snyder, D., Goudarzi, A. & Teuscher, C. Computational capabilities of random automata networks for reservoir computing. *Physical Review E* **87,** 042808 (2013).
7. Chen, T. *et al.* Classification with a disordered dopant-atom network in silicon. *Nature* **577,** 341–345 (2020).
8. Van der Sande, G., Brunner, D. & Soriano, M. C. Advances in photonic reservoir computing. *Nanophotonics* **6,** 561–576 (2017).
9. Torrejon, J. *et al.* Neuromorphic computing with nanoscale spintronic oscillators. *Nature* **547,** 428–431 (2017).
10. Nakajima, K., Hauser, H., Li, T. & Pfeifer, R. Information processing via physical soft body. *Scientific Reports* **5,** 10487 (2015).
11. Didovyk, A. *et al.* Distributed Classifier Based on Genetically Engineered Bacterial Cell Cultures. *ACS Synthetic Biology* **4,** 72–82 (2015).
12. Blokhuis, A., Lacoste, D. & Gaspard, P. Reaction kinetics in open reactors and serial transfers between closed reactors. *The Journal of Chemical Physics* **148,** 144902 (2018).
13. Hopfield, J. J. Neural networks and physical systems with emergent collective computational abilities. *Proceedings of the National Academy of Sciences* **79,** 2554–2558 (1982).
14. Cucchi, M., Abreu, S., Ciccone, G., Brunner, D. & Kleemann, H. Hands-on reservoir computing: a tutorial for practical implementation. en. *Neuromorphic Computing and Engineering* **2,** 032002 (2022).
15. Virtanen, P. *et al.* SciPy 1.0: fundamental algorithms for scientific computing in Python. *Nature Methods* **17,** 261–272 (2020).
16. Boughorbel, S., Jarray, F. & El-Anbari, M. Optimal classifier for imbalanced data using Matthews Correlation Coefficient metric. en. *PLOS ONE* **12,** e0177678 (2017).
17. Oliveira, A. S. F., Ciccotti, G., Haider, S. & Mulholland, A. J. Dynamical nonequilibrium molecular dynamics reveals the structural basis for allostery and signal propagation in biomolecular systems. *The European Physical Journal B* **94** (2021).

18. Fröhlich, F. *et al.* AMICI: high-performance sensitivity analysis for large ordinary differential equation models. *Bioinformatics* **37,** 3676–3677 (2021).

19. Kraskov, A., Stögbauer, H. & Grassberger, P. Estimating mutual information. *Physical Review E* **69,** 066138 (2004).

20. Pedregosa, F. *et al.* Scikit-learn: Machine Learning in Python. *Journal of Machine Learning Research* **12,** 2825–2830 (2011).

# Supplementary Figures

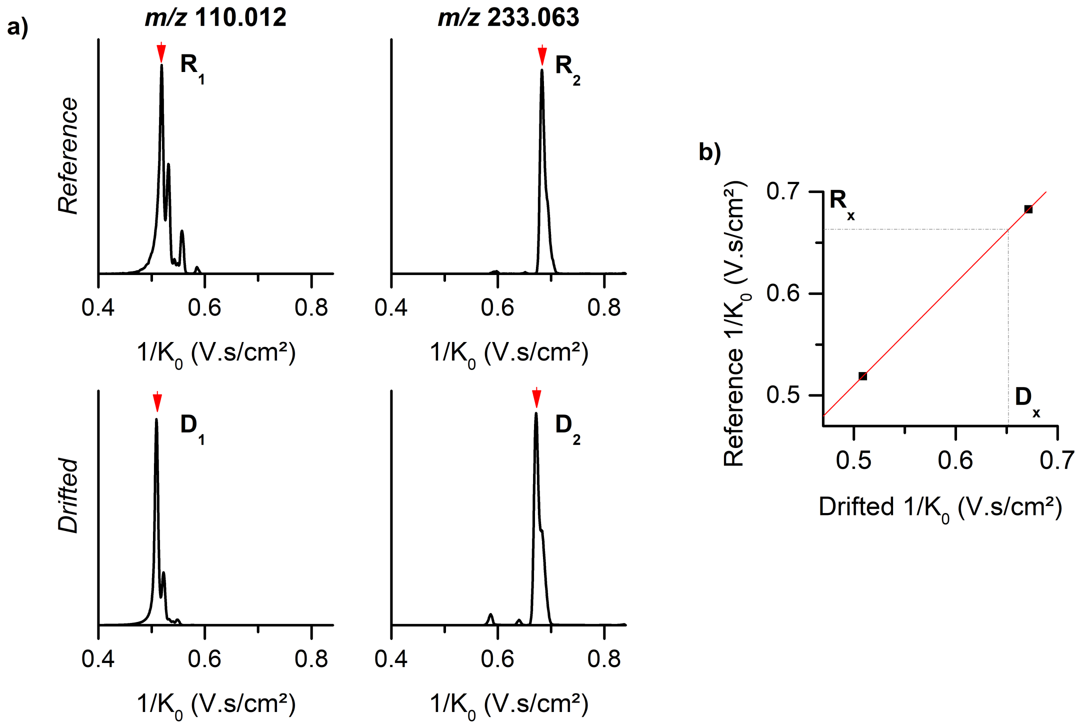


Figure S1: **a)** Comparison of the apex of the mobilograms for two ions, $m/z$ 110.012 ($[C_6H_{12}O_6Ca]^{2+}$) and 233.063 ($[C_7H_{14}O_7Na]^{+}$), between a reference measurement ($R_1$ and $R_2$) and a measurement where a drift is observed ($D_1$ and $D_2$). **b)** Any reference inverse mobility ($R_x$) can be adjusted for drift by using the equation 1. The corresponding drifted value is named $D_x$. For each experiment, reference inverse mobilities included in the peak table are adjusted for drift, thereby ensuring that similar peaks are processed in different experiments regardless of any drift.

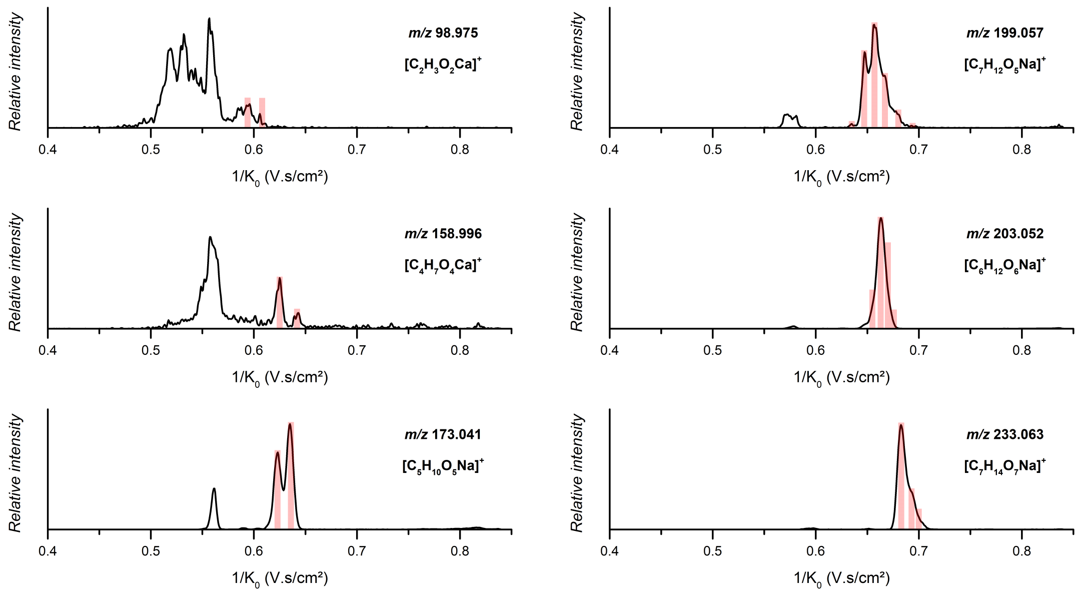


Figure S2: Selected singly charged ions considered for data. Selected inverse mobility ranges are highlighted by red rectangles. The inverse mobility ranges not considered were attributed to isobaric doubly charged ions, as evidenced by their isotopic pattern.

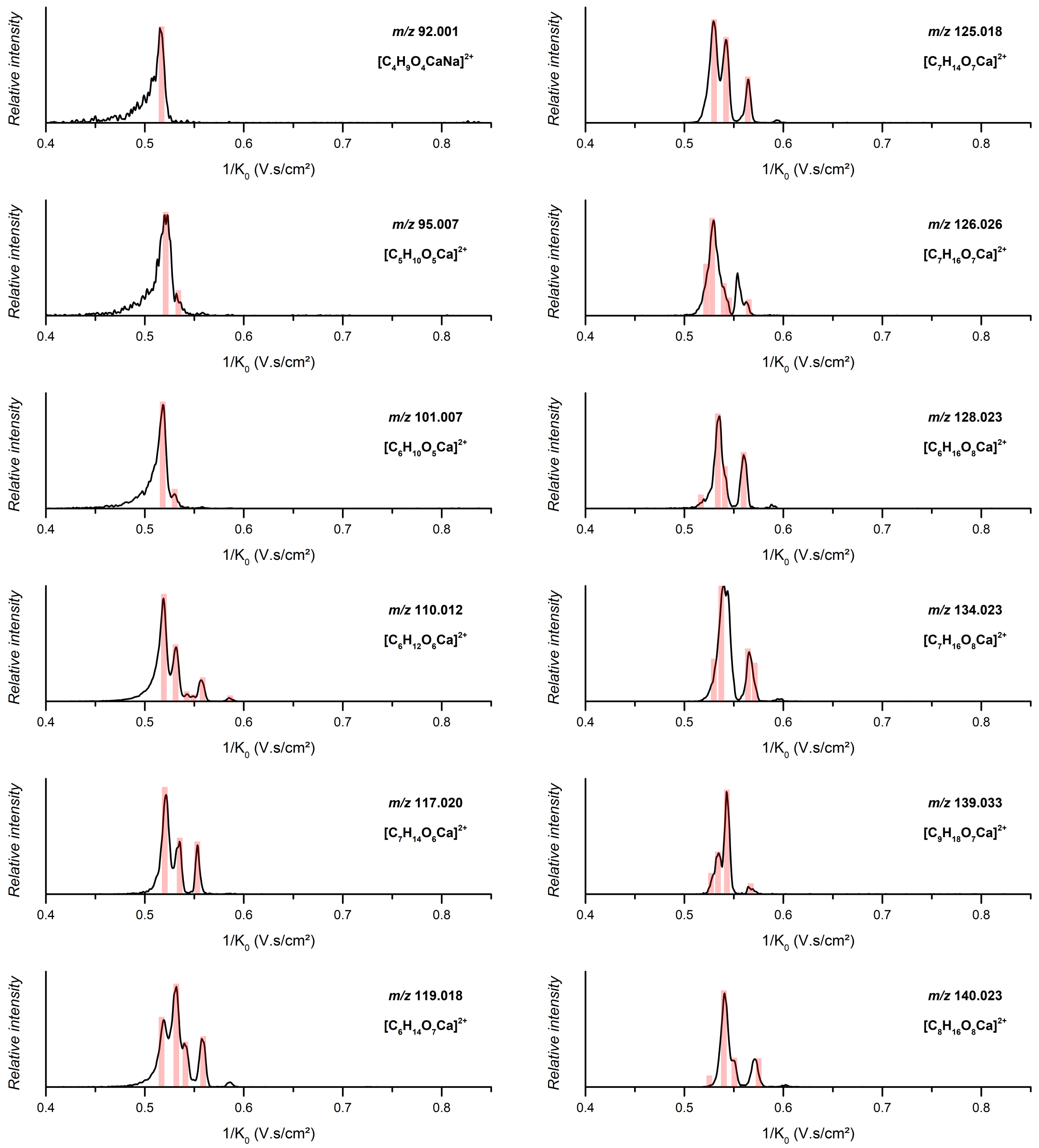


Figure S3: Selected doubly charged ions considered for data extraction. Selected inverse mobility ranges are highlighted by red rectangles.

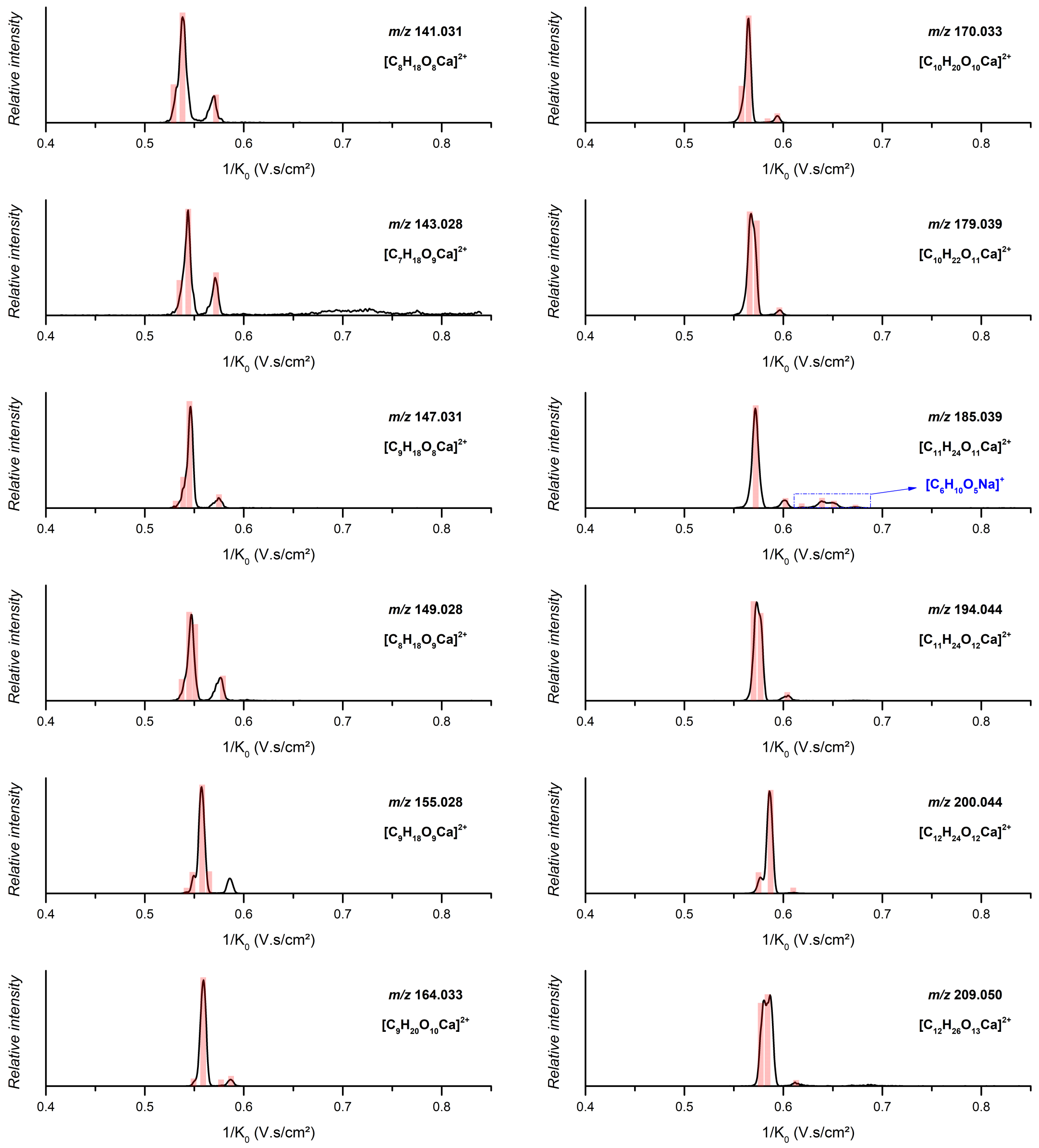


Figure S4: Selected doubly charged ions considered for data extraction. Selected inverse mobility ranges are highlighted by red rectangles.

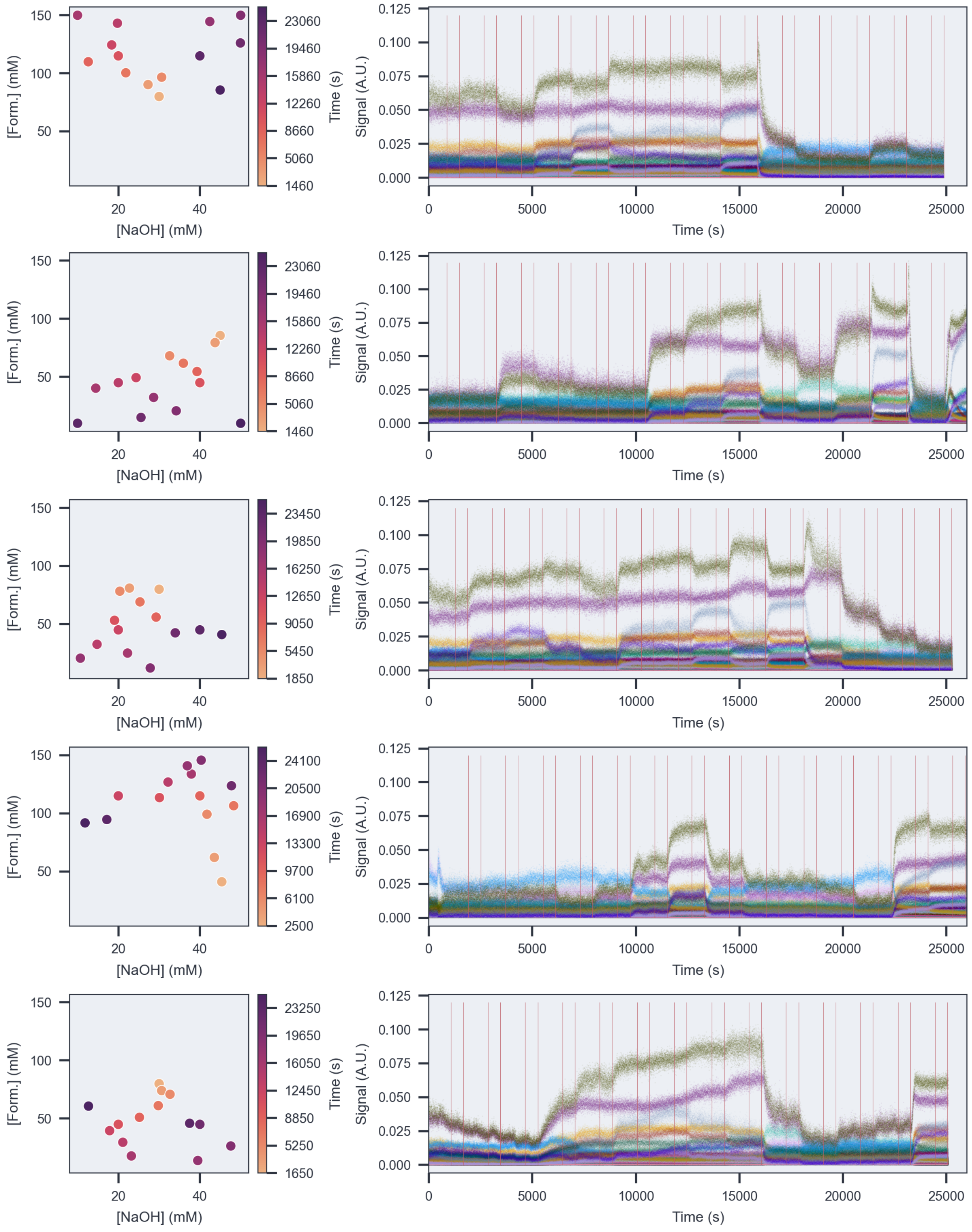


Figure S5: Steady state inputs and reservoir response for multiple experiments and measurement runs. On the left, the inputs per experiment are shown with colour indicating the order. On the right, the measured ion signals in response to the different inputs are shown. Areas indicated between vertical bars are used as steady state data.

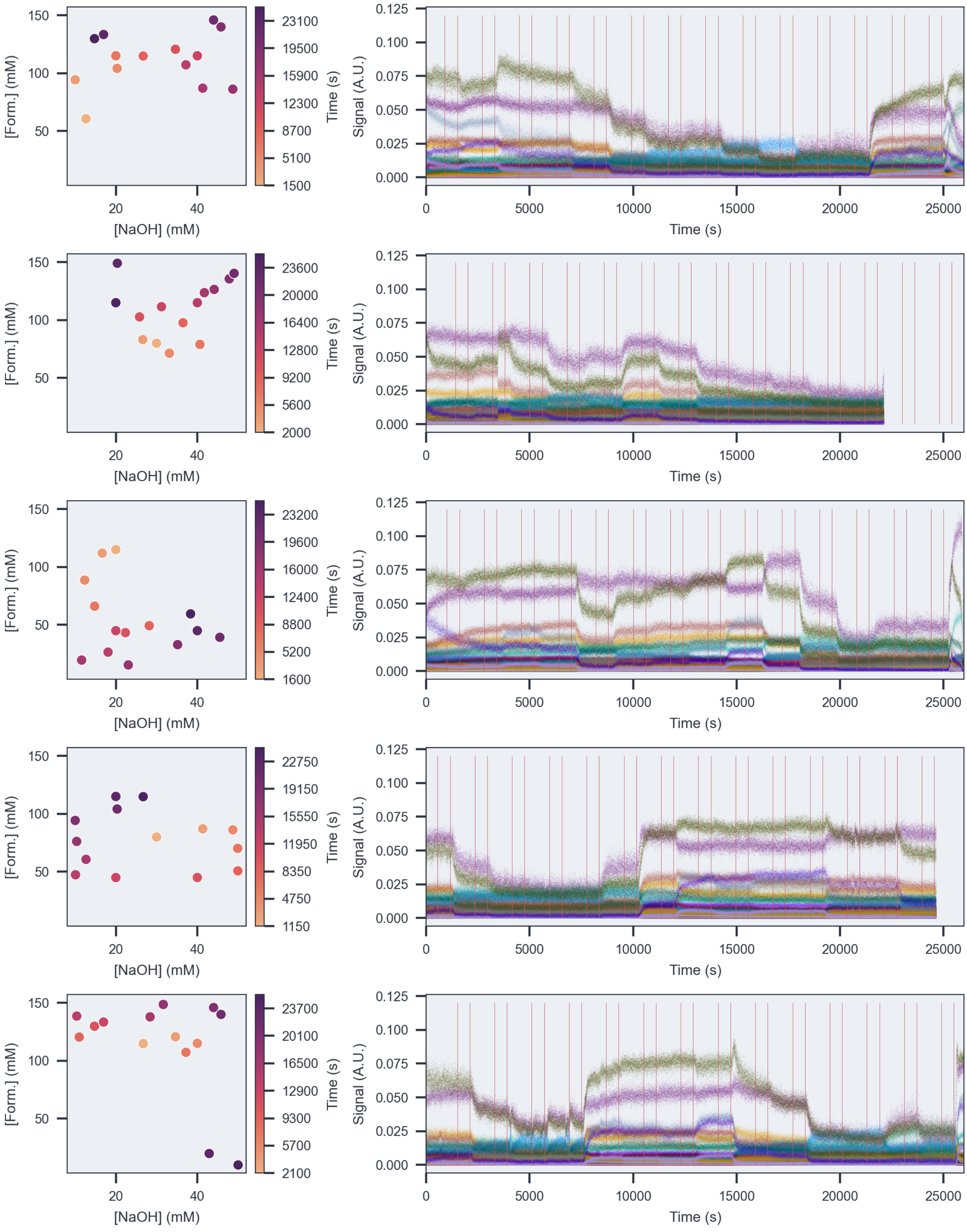


Figure S6: Steady state inputs and reservoir response for multiple experiments and measurement runs. On the left, the inputs per experiment are shown with colour indicating the order. On the right, the measured ion signals in response to the different inputs are shown. Areas indicated between vertical bars are used as steady state data.

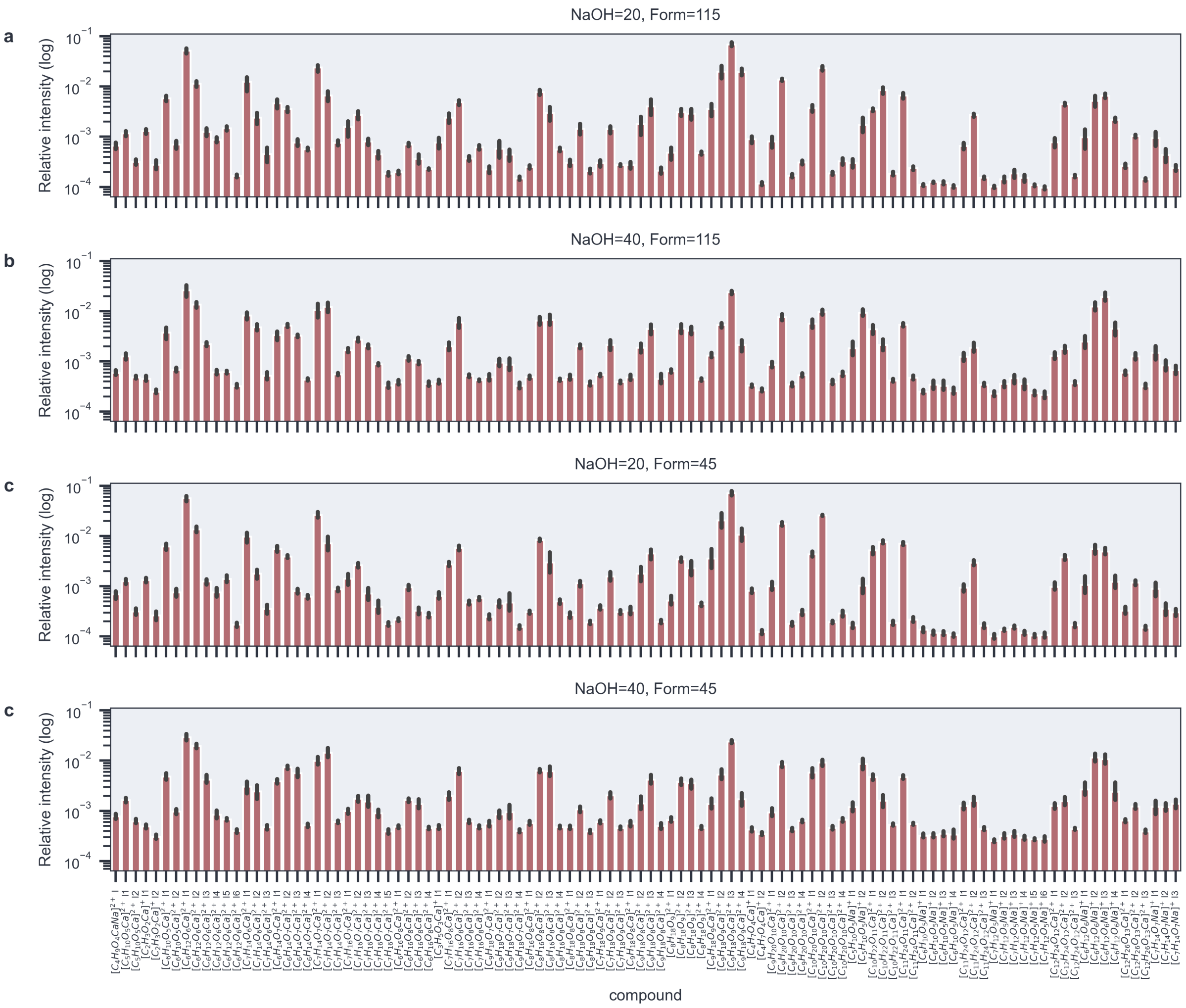


Figure S7: Bar plots showing the reproducibility of ion intensities measured by mass spectrometry for every compound of interest, across four different days, for identical input conditions. Average intensities are represented by the bars, standard deviations in the intensities by the black error bars. a) 20mM NaOH, 115mM b) 40mM NaOH, 115mM c) 20mM NaOH, 45mM d) 40mM NaOH, 115 mM formaldehyde.

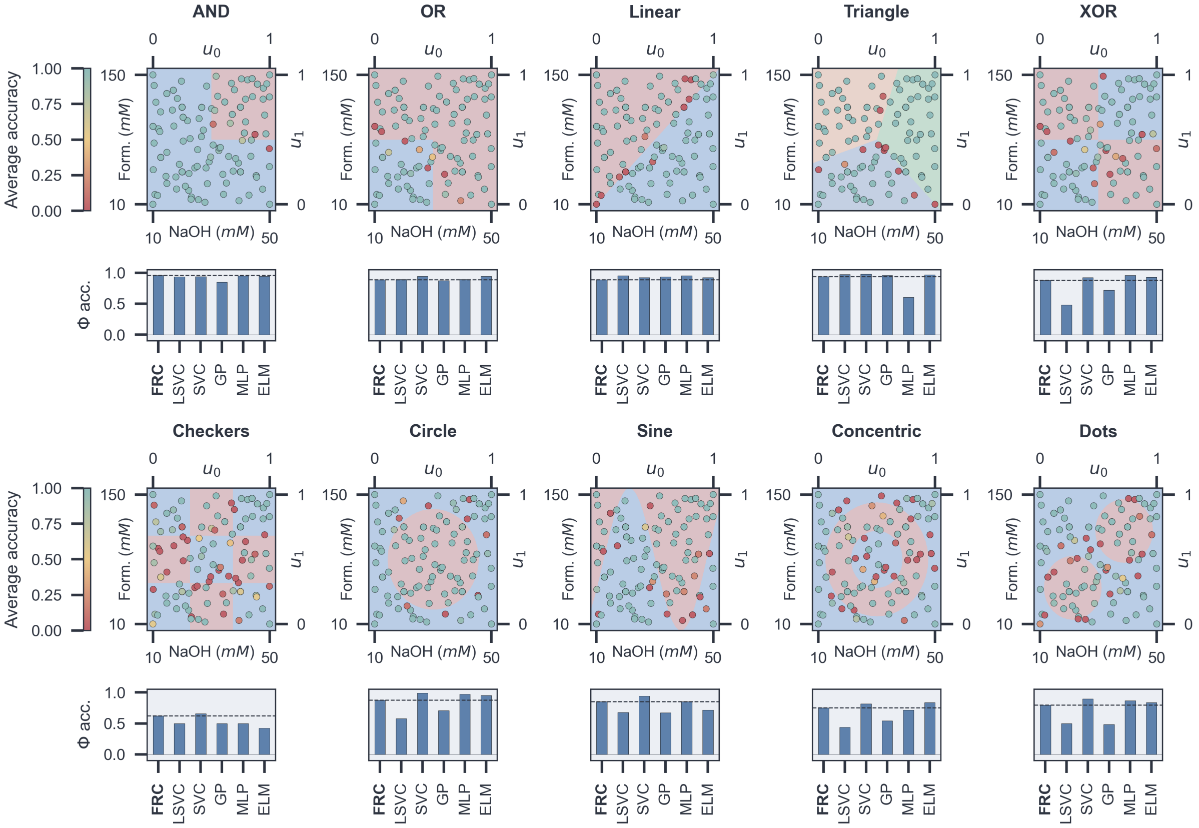


Figure S8: Scatter plots showing for every classification task the classification accuracy per input, averaged over 100 random train-test splits, obtained from a Lasso regression.

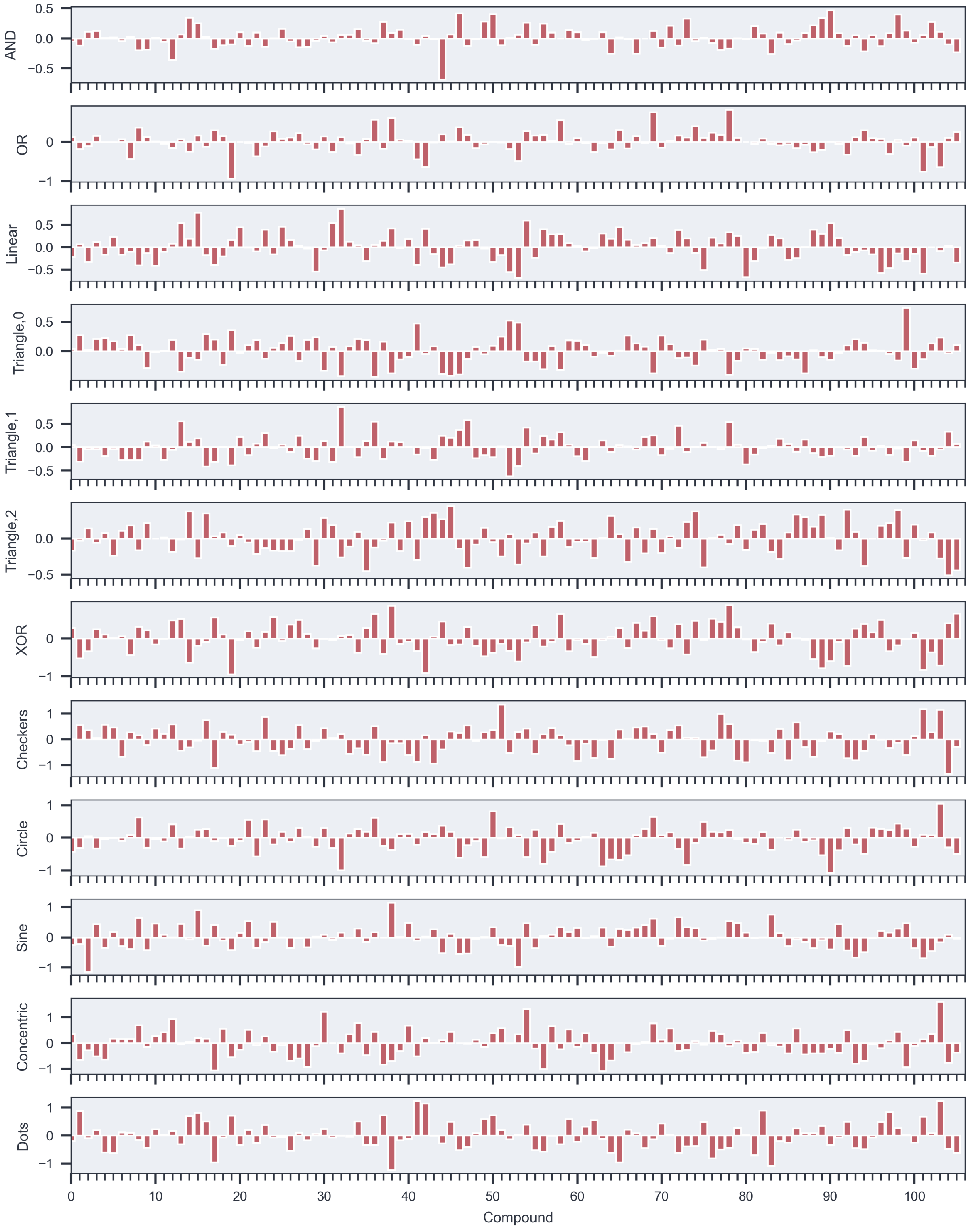


Figure S9: Weights obtained from the Ridge regression training of various tasks, on a normalized data set. Weights are represented by a vertical bar per compound, with compounds numbered according to table 1

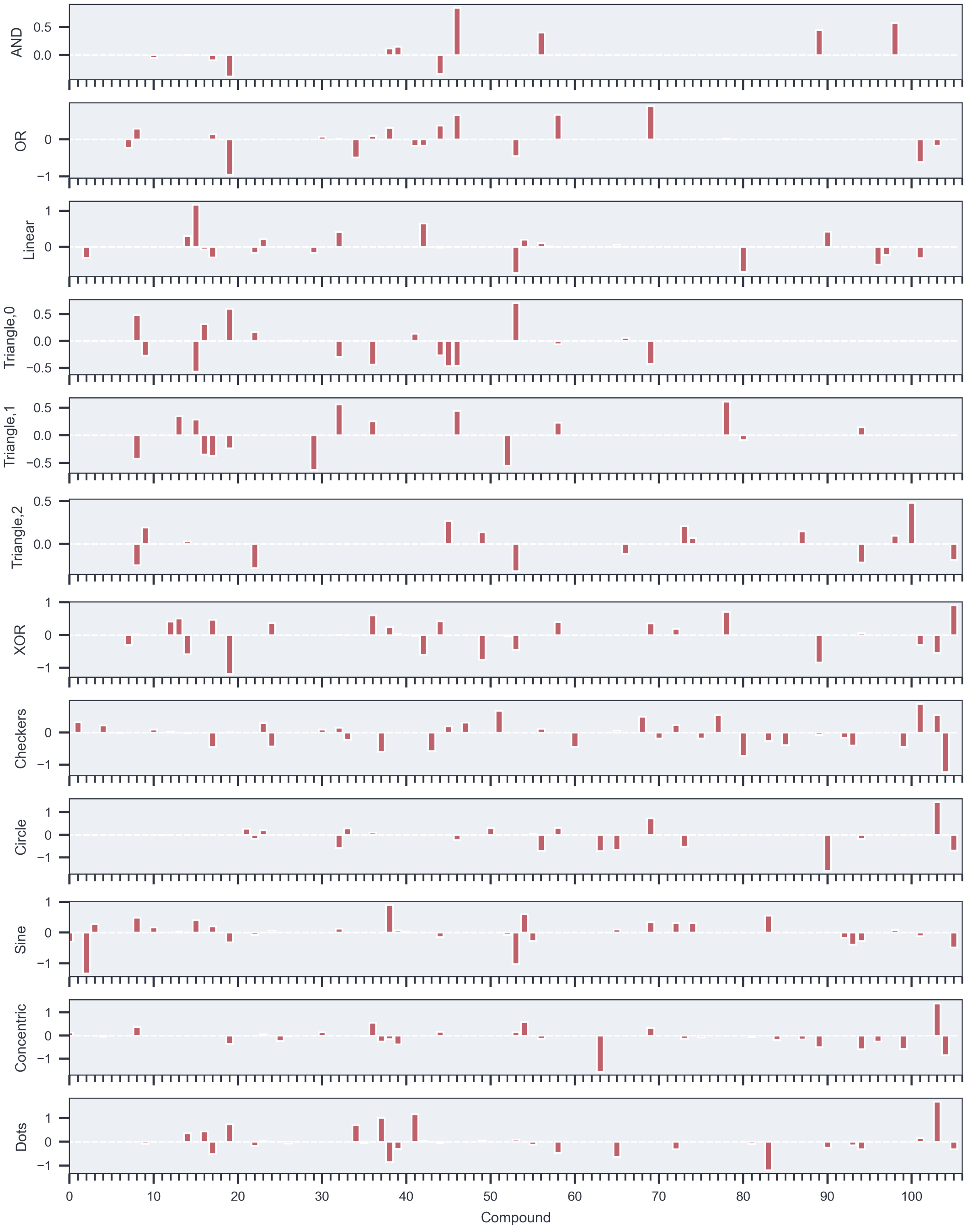


Figure S10: Weights obtained from the Lasso regression training of various tasks, on a normalized data set. Weights are represented by a vertical bar per compound, with compounds numbered according to table 1

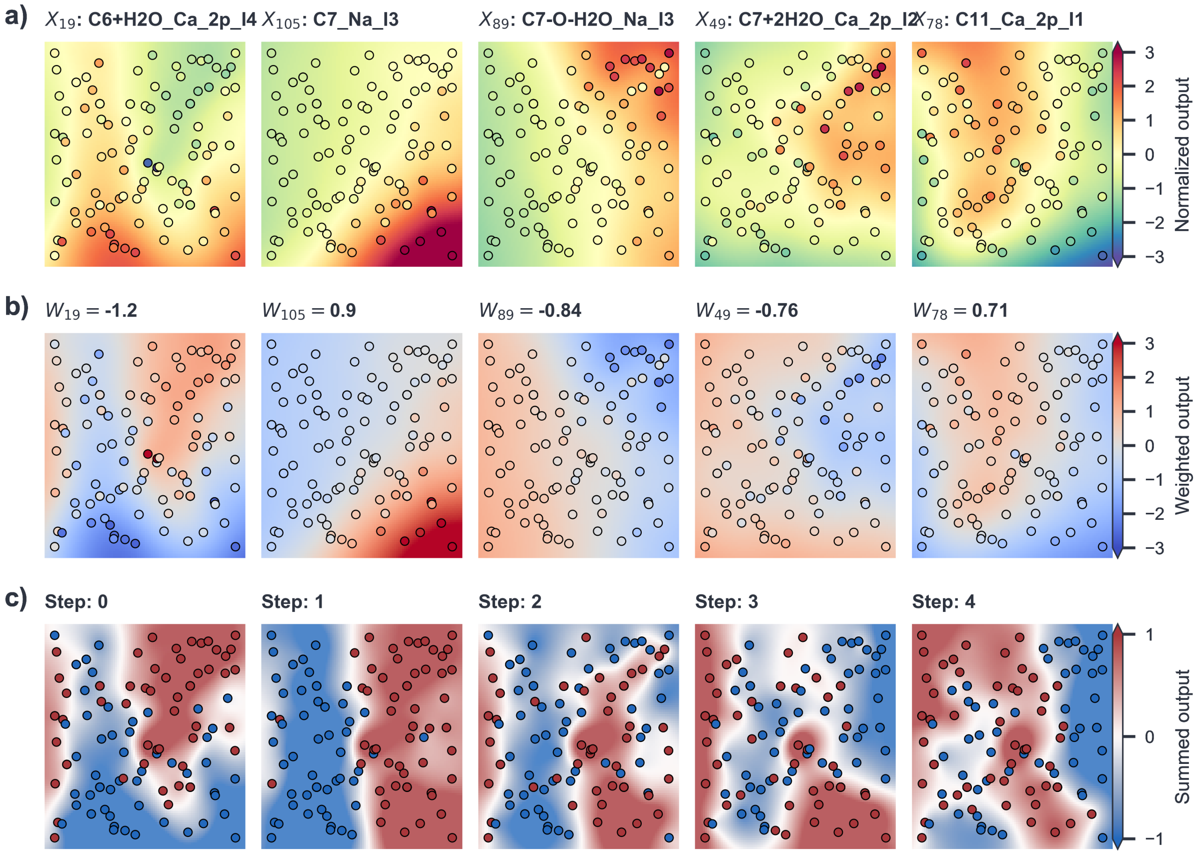


Figure S11: (1 of 5) Visualization of the creation of an XOR gate by composition of different compounds with different weights as obtained through a lasso regression, ordered by the absolute value of weights. Every fifth compounds is shown here for brevity, a full version of 25 compounds is included in the Supporting Information. a) Plots showing measured intensities for a compound (normalized to standard scores) and a shaded background obtained by interpolation. Plots are sorted by decreasing absolute weight value (contribution in the classification process). Axis are the same as in S8, with NaOH input on the x-axis and formaldehyde input on the y-axis. b) Plots showing the same intensities as in the top row, now multiplied by the weight for that respective compound. c) Plots showing the resulting classification by weighting and summing up contributions from all compounds included at that point.

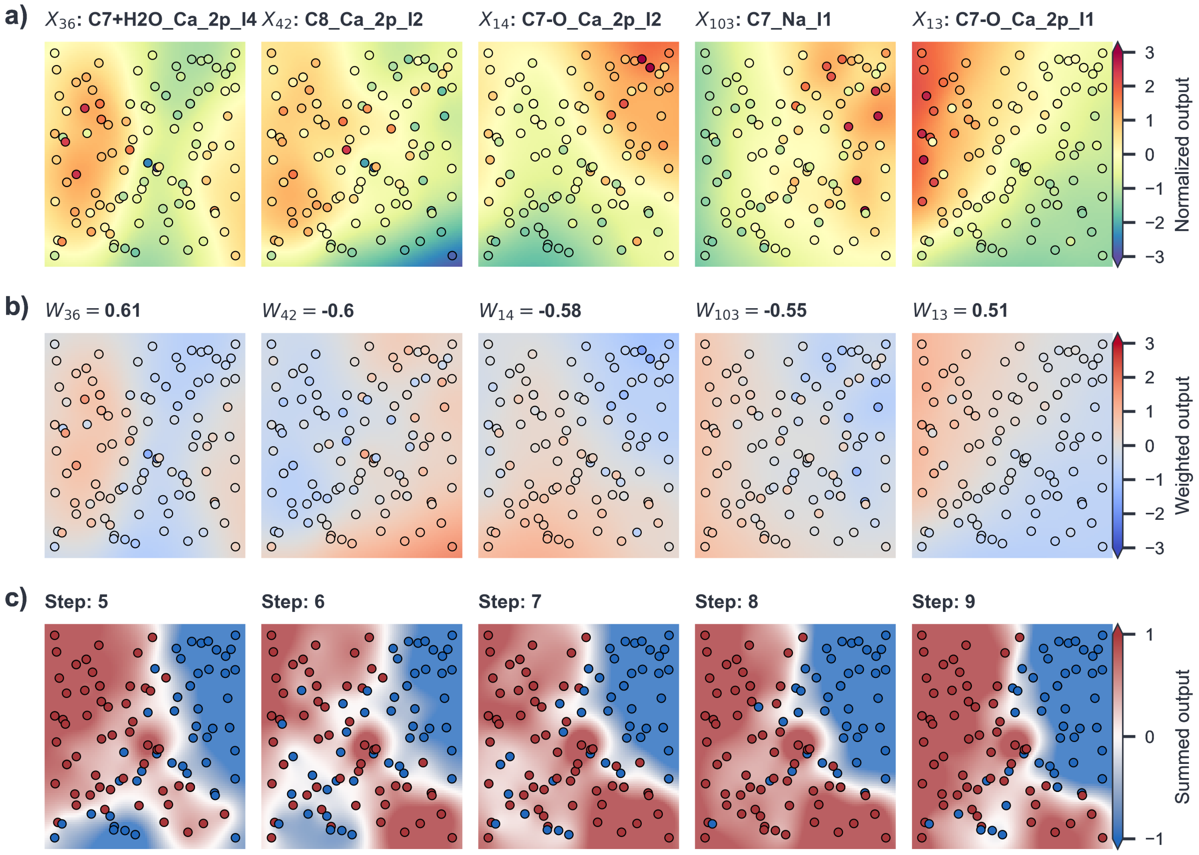


Figure S12: (2 of 5) Visualization of the creation of an XOR gate by composition of different compounds with different weights as obtained through a lasso regression, ordered by the absolute value of weights. Every fifth compounds is shown here for brevity, a full version of 25 compounds is included in the Supporting Information. a) Plots showing measured intensities for a compound (normalized to standard scores) and a shaded background obtained by interpolation. Plots are sorted by decreasing absolute weight value (contribution in the classification process). Axis are the same as in S8, with NaOH input on the x-axis and formaldehyde input on the y-axis. b) Plots showing the same intensities as in the top row, now multiplied by the weight for that respective compound. c) Plots showing the resulting classification by weighting and summing up contributions from all compounds included at that point.

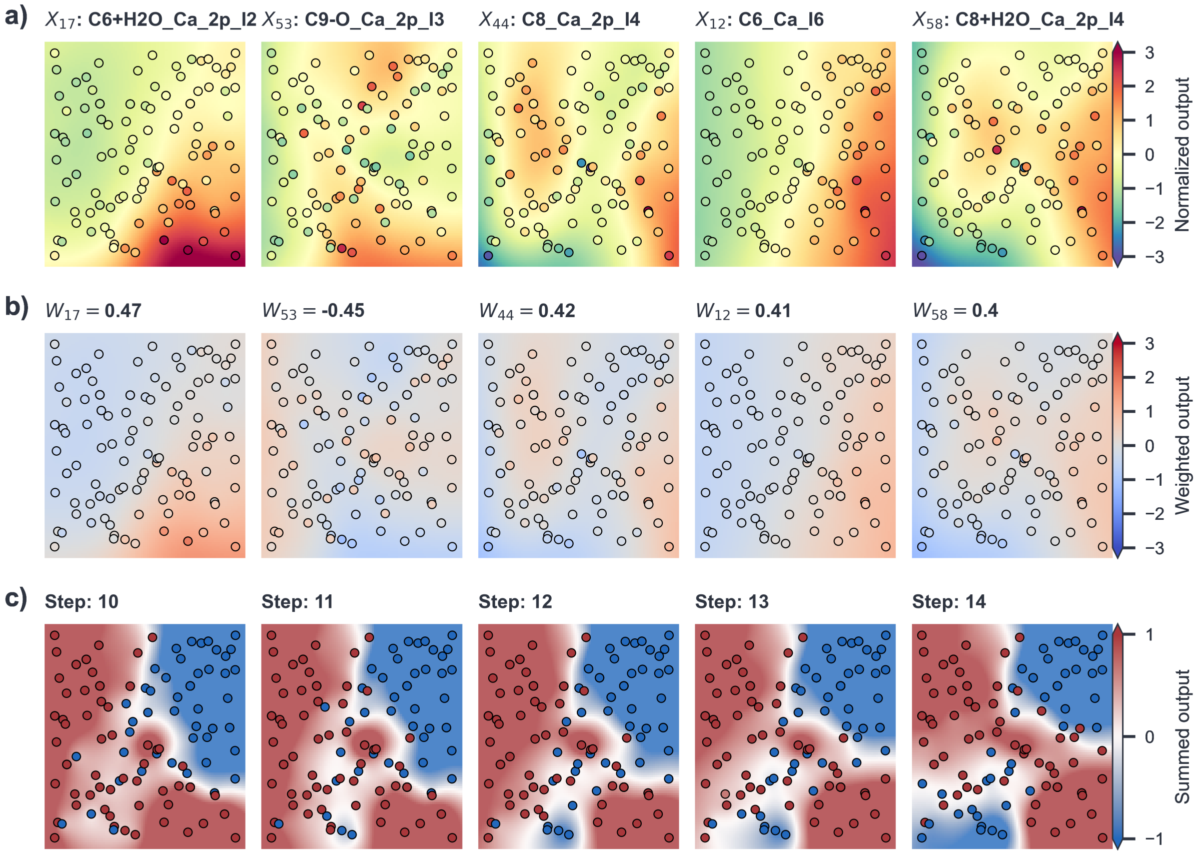


Figure S13: (3 of 5) Visualization of the creation of an XOR gate by composition of different compounds with different weights as obtained through a lasso regression, ordered by the absolute value of weights. Every fifth compounds is shown here for brevity, a full version of 25 compounds is included in the Supporting Information. a) Plots showing measured intensities for a compound (normalized to standard scores) and a shaded background obtained by interpolation. Plots are sorted by decreasing absolute weight value (contribution in the classification process). Axis are the same as in S8, with NaOH input on the x-axis and formaldehyde input on the y-axis. b) Plots showing the same intensities as in the top row, now multiplied by the weight for that respective compound. c) Plots showing the resulting classification by weighting and summing up contributions from all compounds included at that point.

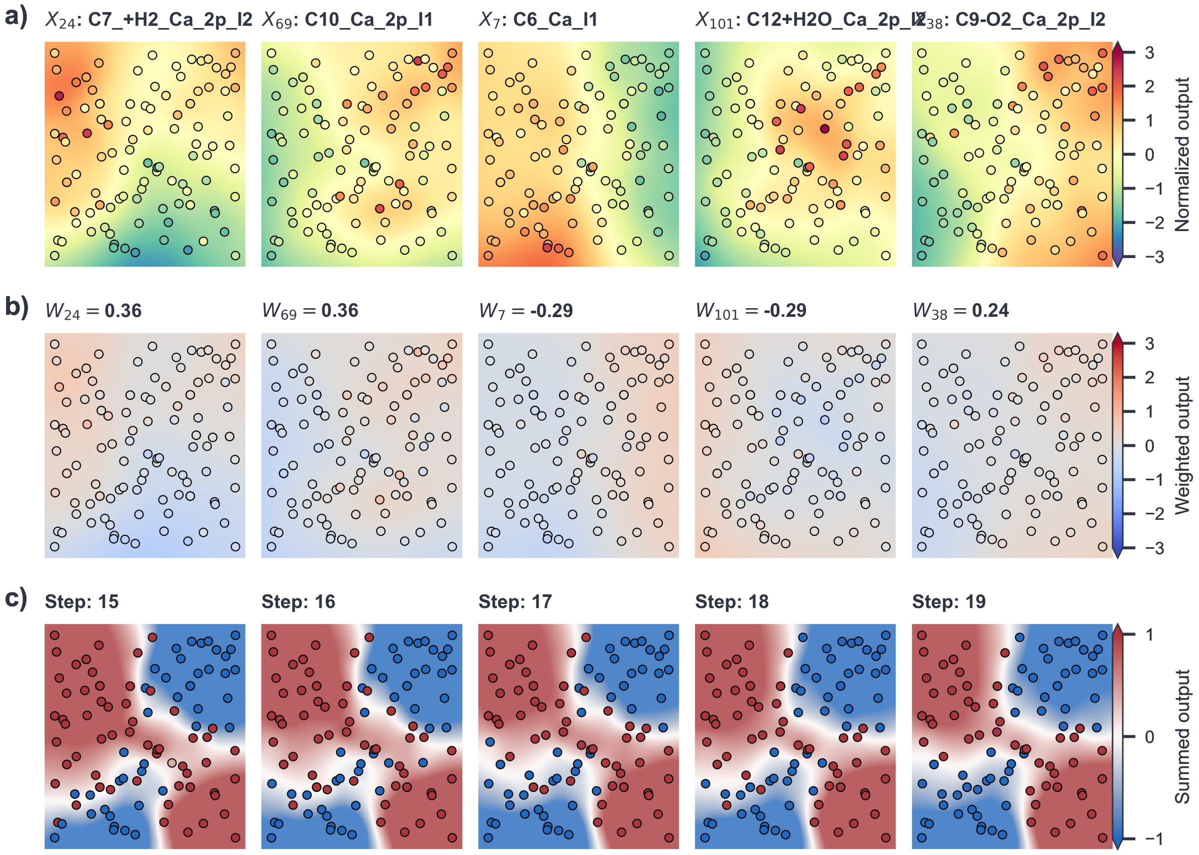


Figure S14: (4 of 5) Visualization of the creation of an XOR gate by composition of different compounds with different weights as obtained through a lasso regression, ordered by the absolute value of weights. Every fifth compounds is shown here for brevity, a full version of 25 compounds is included in the Supporting Information. a) Plots showing measured intensities for a compound (normalized to standard scores) and a shaded background obtained by interpolation. Plots are sorted by decreasing absolute weight value (contribution in the classification process). Axis are the same as in S8, with NaOH input on the x-axis and formaldehyde input on the y-axis. b) Plots showing the same intensities as in the top row, now multiplied by the weight for that respective compound. c) Plots showing the resulting classification by weighting and summing up contributions from all compounds included at that point.

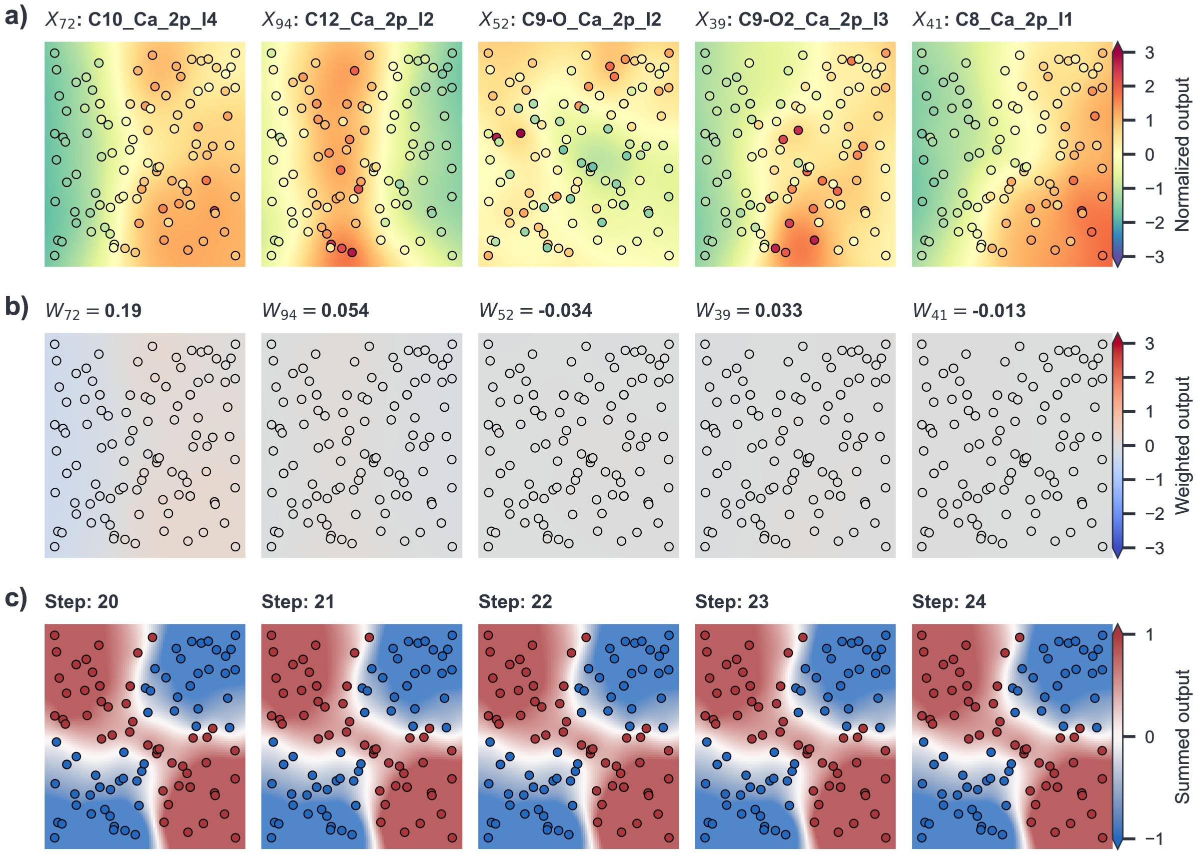


Figure S15: (5 of 5) Visualization of the creation of an XOR gate by composition of different compounds with different weights as obtained through a lasso regression, ordered by the absolute value of weights. Every fifth compounds is shown here for brevity, a full version of 25 compounds is included in the Supporting Information. a) Plots showing measured intensities for a compound (normalized to standard scores) and a shaded background obtained by interpolation. Plots are sorted by decreasing absolute weight value (contribution in the classification process). Axis are the same as in S8, with NaOH input on the x-axis and formaldehyde input on the y-axis. b) Plots showing the same intensities as in the top row, now multiplied by the weight for that respective compound. c) Plots showing the resulting classification by weighting and summing up contributions from all compounds included at that point.

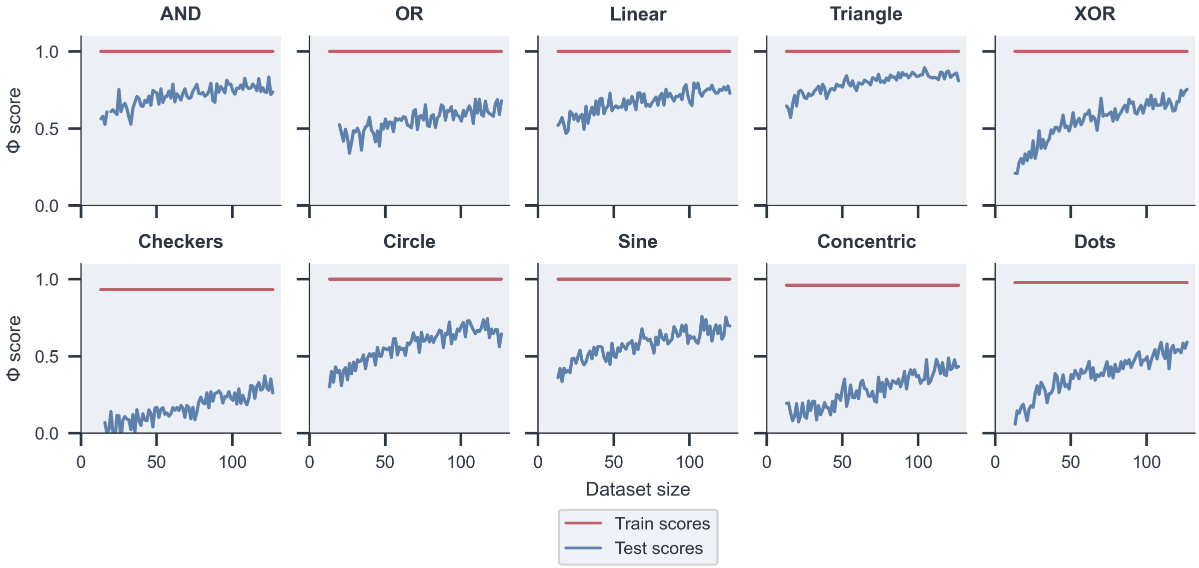


Figure S16: Training curves for classification tasks as a function of the train-set size, where all samples not in the train-set are used for the test-set. Scores are calculated using the $\Phi$ coefficient. Training scores are indicated in orange, test scores are indicated in blue.

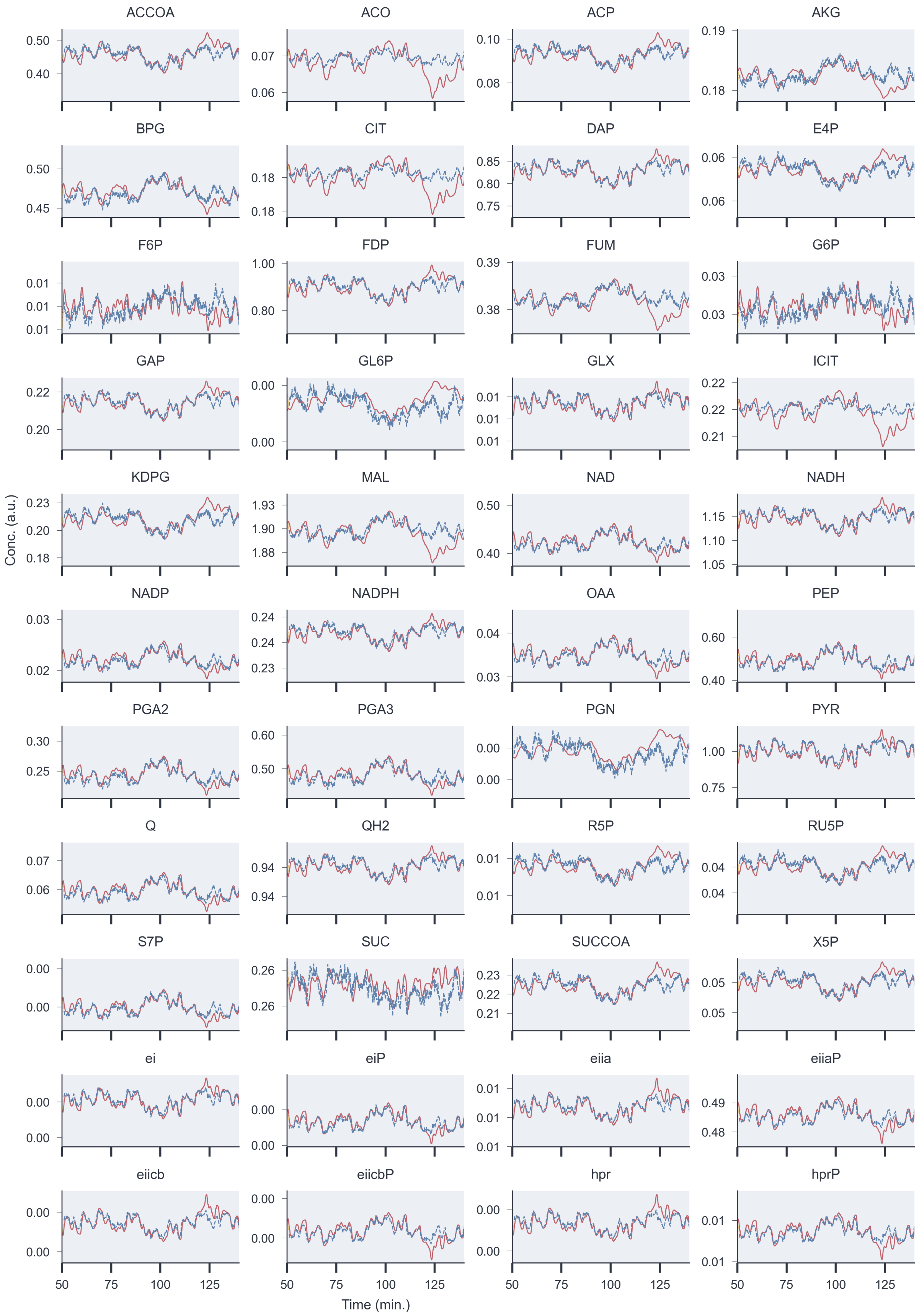


Figure S17: Prediction results for substrates in the metabolic network

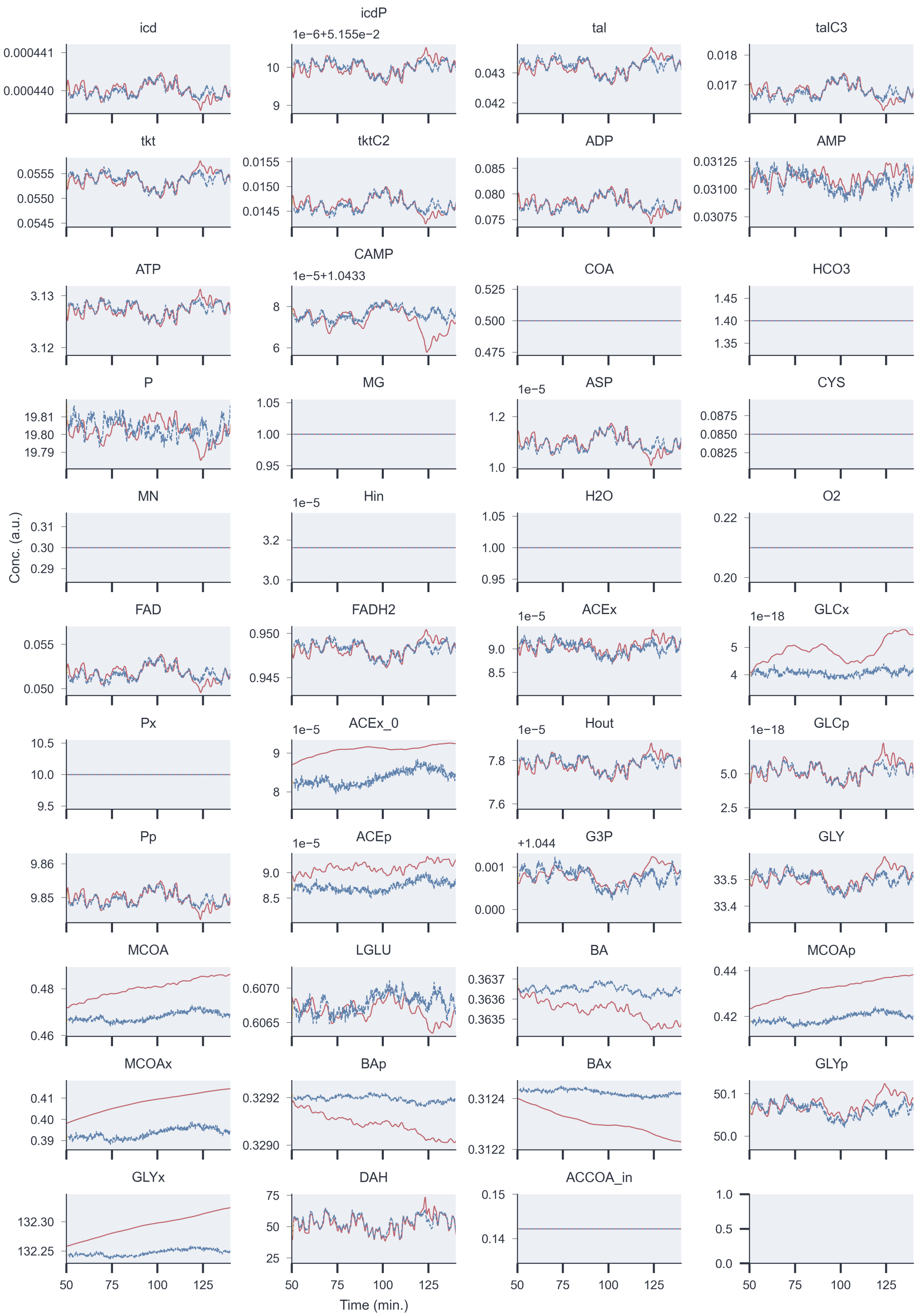


Figure S18: Prediction results for substrates in the metabolic network (cont.)

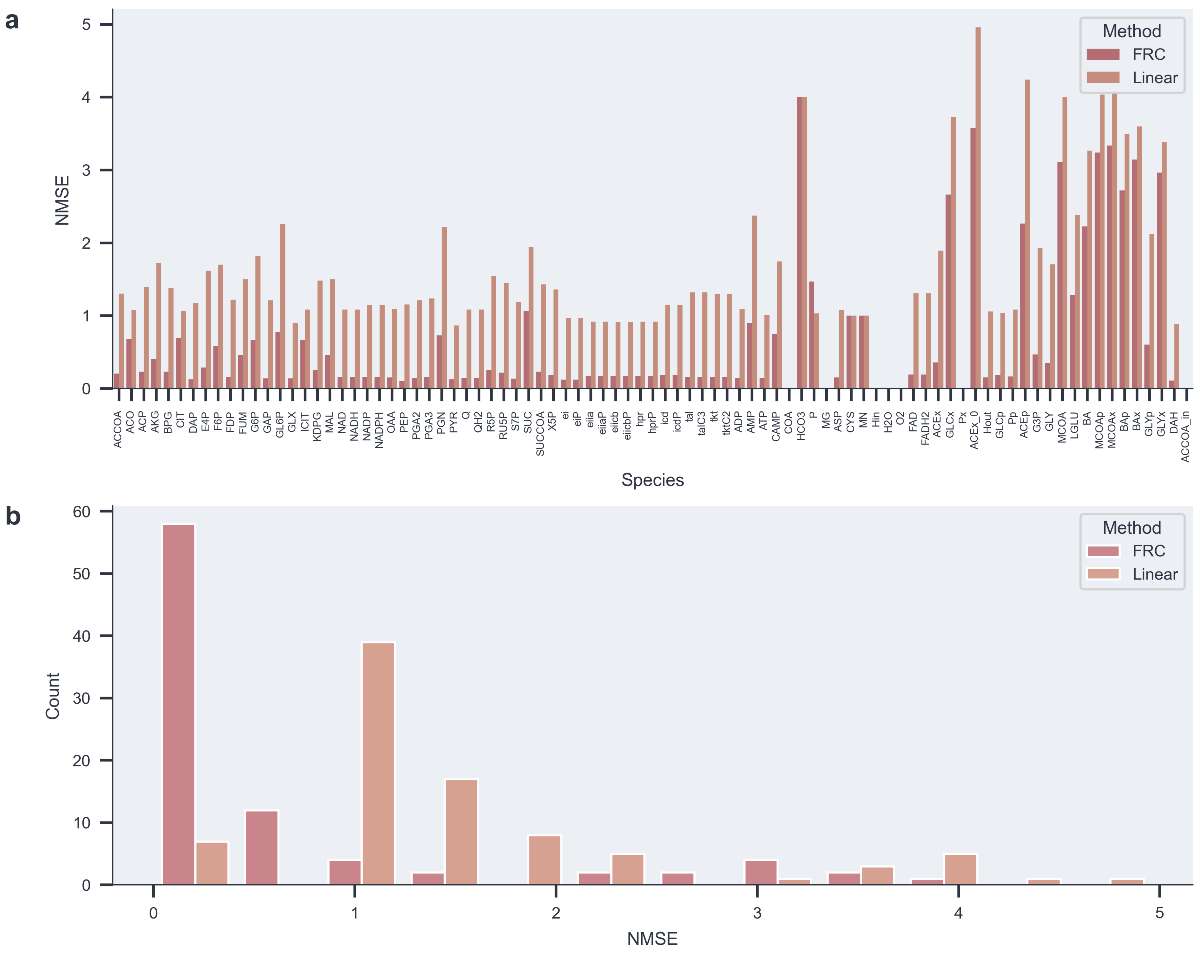


Figure S19: a) Normalized mean-squared error (NMSE) per substrate in the metabolic network, both for predictions made by the Formose Reservoir (FRC) and a linear, Ridge regression predictor. b) Histogram of NMSE scores for both prediction methods

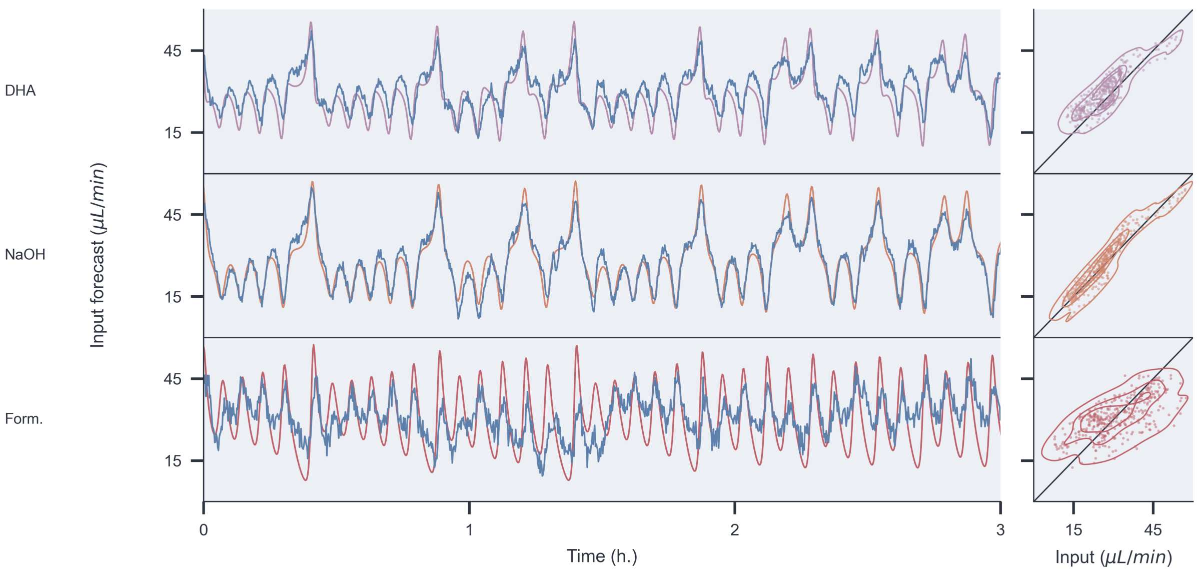


Figure S20: Extended time traces and comparison plots for 2-minute forecasts of simultaneously varying DHA, NaOH and formaldehyde inputs that resemble the behaviour of a Lorenz attractor. True inputs are shown as purple, orange and red lines respectively, and the forecasts as blue lines.

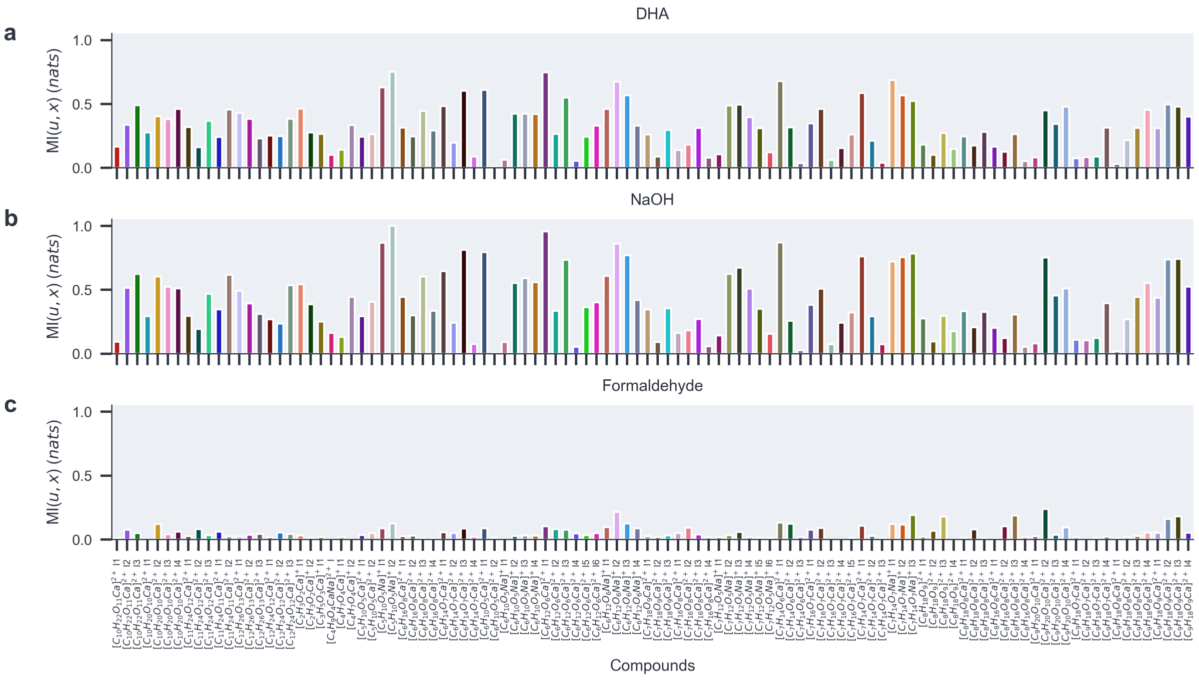


Figure S21: Direct mutual information between Lorenz attractor DHA, NaOH and formaldehyde inputs and ion signal output. Every bar corresponds to the mutual information in units of nats per compound, calculated over the full duration of the experiment.
